# Short-separation regression incorporated diffuse optical tomography image reconstruction modeling for high-density functional near-infrared spectroscopy

**DOI:** 10.1117/1.NPh.10.2.025007

**Published:** 2023-05-23

**Authors:** Yuanyuan Gao, De’Ja Rogers, Alexander von Lühmann, Antonio Ortega-Martinez, David A. Boas, Meryem Ayşe Yücel

**Affiliations:** Boston University, Neurophotonics Center, Boston, Massachusetts, United States

**Keywords:** high-density functional near-infrared spectroscopy, diffuse optical tomography, short-separation regression, optical image reconstruction

## Abstract

**Significance:**

Short-separation (SS) regression and diffuse optical tomography (DOT) image reconstruction, two widely adopted methods in functional near-infrared spectroscopy (fNIRS), were demonstrated to individually facilitate the separation of brain activation and physiological signals, with further improvement using both sequentially. We hypothesized that doing both simultaneously would further improve the performance.

**Aim:**

Motivated by the success of these two approaches, we propose a method, SS-DOT, which applies SS and DOT simultaneously.

**Approach:**

The method, which employs spatial and temporal basis functions to represent the hemoglobin concentration changes, enables us to incorporate SS regressors into the time series DOT model. To benchmark the performance of the SS-DOT model against conventional sequential models, we use fNIRS resting state data augmented with synthetic brain response as well as data acquired during a ball squeezing task. The conventional sequential models comprise performing SS regression and DOT.

**Results:**

The results show that the SS-DOT model improves the image quality by increasing the contrast-to-background ratio by a threefold improvement. The benefits are marginal at small brain activation.

**Conclusions:**

The SS-DOT model improves the fNIRS image reconstruction quality.

## 1 Introduction

Continuous-wave functional near-infrared spectroscopy (fNIRS) is a noninvasive neuroimaging technique that measures cerebral hemoglobin concentration changes using an array of light sources and detectors placed on the surface of the head.^1^^–^^5^ The detected light reflects the hemodynamic changes not only in the cortex but also in the superficial tissues through which the light propagates, i.e., scalp, skull, and cerebrospinal fluid (CSF).^6^^–^^9^ Moreover, measurements are more sensitive to extracerebral tissues that are closer to the detectors and much less sensitive to the brain cortex, where the brain functional activity takes place.^10^ Thus accurately modeling the cerebral hemodynamic response function (HRF) and distinguishing it from the extracerebral signals is essential to robustly estimating brain activation.

Various methods to filter the systemic interference from the fNIRS measurement have been described in the literature. Low-pass filtering is widely used as it is highly effective at removing high-frequency components in the signal such as cardiac oscillations.^4^^,^^11^ However, it tends to overcorrect the signal when the brain signals have a significant overlap in the frequency spectrum with other physiological variations, such as respiration or Mayer waves.^12^ As such, more powerful methods for global noise reduction have been developed. For example, auxiliary physiological measurements are regressed out from fNIRS measurements, such as heart beat,^13^^–^^15^ blood pressure and heart rate,^15^^–^^17^ scalp hemodynamic changes obtained by fMRI,^18^ and another source–detector measurement performed over a nonactivated region of the brain.^4^ Data-driven methods were also used to reduce global noise, such as principal component analysis^19^^,^^20^ and independent component analysis.^21^

Beyond the previously mentioned methods, two fundamental approaches have been suggested: (1) inclusion of short-separation (SS) measurements as a systemic physiological regressor and (2) high-density diffuse optical tomography (DOT) to spatially resolve brain activity from physiological contamination in the scalp. The SS measurement approach^22^^–^^27^ utilizes the fact that long-separation channel (∼30  mm) signals sample both cerebral and extracerebral tissues and SS channel (∼8  mm) signals mostly sample the extracerebral tissues. The signal measured with the SS channels, representing mostly the extracerebral hemodynamics, can be regressed out from the signal measured at the long-separation channels, improving the estimation of cerebral hemodynamics. Moreover, some of the systemic physiological interference arising from cardiac activity, respiration, and other homeostatic processes that appear both in cerebral and extracerebral layers can also be captured by the SS measurements and can be regressed out from the long separation measurements. Two widely used methods for the estimation of the HRF are the linear least square estimator/general linear model (GLM) approach^22^^,^^27^^–^^29^ and the adaptive filtering technique that updates the coefficients at each time point.^23^^,^^27^^,^^30^

DOT reconstructs three-dimensional (3D) maps of the hemoglobin concentration changes.^3^^,^^7^^,^^9^^,^^31^ It is usually used on datasets collected with a high-density probe consisting of a dense array of sources and detectors that provide overlapping measurements typically with channels consisting of multiple distances.^9^ The overlapping measurement can increase both the spatial resolution and the depth resolution. The disentanglement of extracerebral and cerebral hemodynamics is realized by the varying depth sensitivity of the measurements with different source–detector distances, as long-distance channels have more relative sensitivity to the deeper cerebral layer and the short-distance channels are more sensitive to the extracerebral layers. In contrast to the SS regression approach, which is typically applied in channel space, DOT image reconstruction results in an image of the hemoglobin concentration changes. DOT image reconstruction technique has evolved in the past 20 years,^9^^,^^32^^–^^34^ decreasing the localization error and improving the effective resolution of the images.^35^ To construct the DOT forward model, finite-element^36^^–^^38^ or Monte Carlo methods^39^^–^^42^ can be employed. To solve the inverse problem, the regularization priors have also been optimized for DOT, such as depth sensitivity regularization,^43^ singular value decomposition regularization,^44^ and regularization methods adapted from EEG source reconstruction like linear constrained minimum variance beamforming.^45^

One can either estimate the HRF in channel space and reconstruct a brain map using the resultant HRF amplitudes^46^^,^^47^ or first reconstruct an image time series from the fNIRS channel data and then estimate the HRF in image space.^9^^,^^48^ Gregg et al.^26^ tested the performance of implementing SS and DOT approaches by applying them sequentially. The contrast-to-noise ratio was increased, and the image variance across blocks was decreased with either one of the methods, but the best performance was achieved when sequentially combining the two. Simultaneously reconstructing the image and estimating the HRF have also been suggested in the literature.^17^^,^^49^^,^^50^ This approach uses temporal and spatial basis functions to simultaneously model HRF over time and space. The model is flexible and can incorporate auxiliary inputs as regressors, such as arterial blood pressure, respiration, or motion. Motivated by these works, we proposed merging the SS and DOT models into one model and solving it simultaneously. We hypothesize that it would further improve the contrast-to-background ratio (CBR), t-value, and lateralization index.

In this work, we establish a DOT model that incorporates SS regression (SS-DOT) to reconstruct brain activation maps. We design a high-density probe, by which the spatial and temporal basis functions enable simultaneous spatial and temporal reconstructions of the brain activation. We employ Monte Carlo simulation to derive the sensitivity matrix. We then construct the design matrix to comprise brain activation, extracerebral stimulus activation, SS regressors, and drift regressors to set up the forward and inverse models. Using data from simulation and experimental studies, we test SS-DOT against the conventional sequential models, i.e., performing SS regression first and then the DOT image reconstruction. We quantify the performance of each model using the metrics of CBR, t-value, and lateralization index.

## 2 Methods

### 2.1 High-Density Probe Layout

**Fig. 1:**
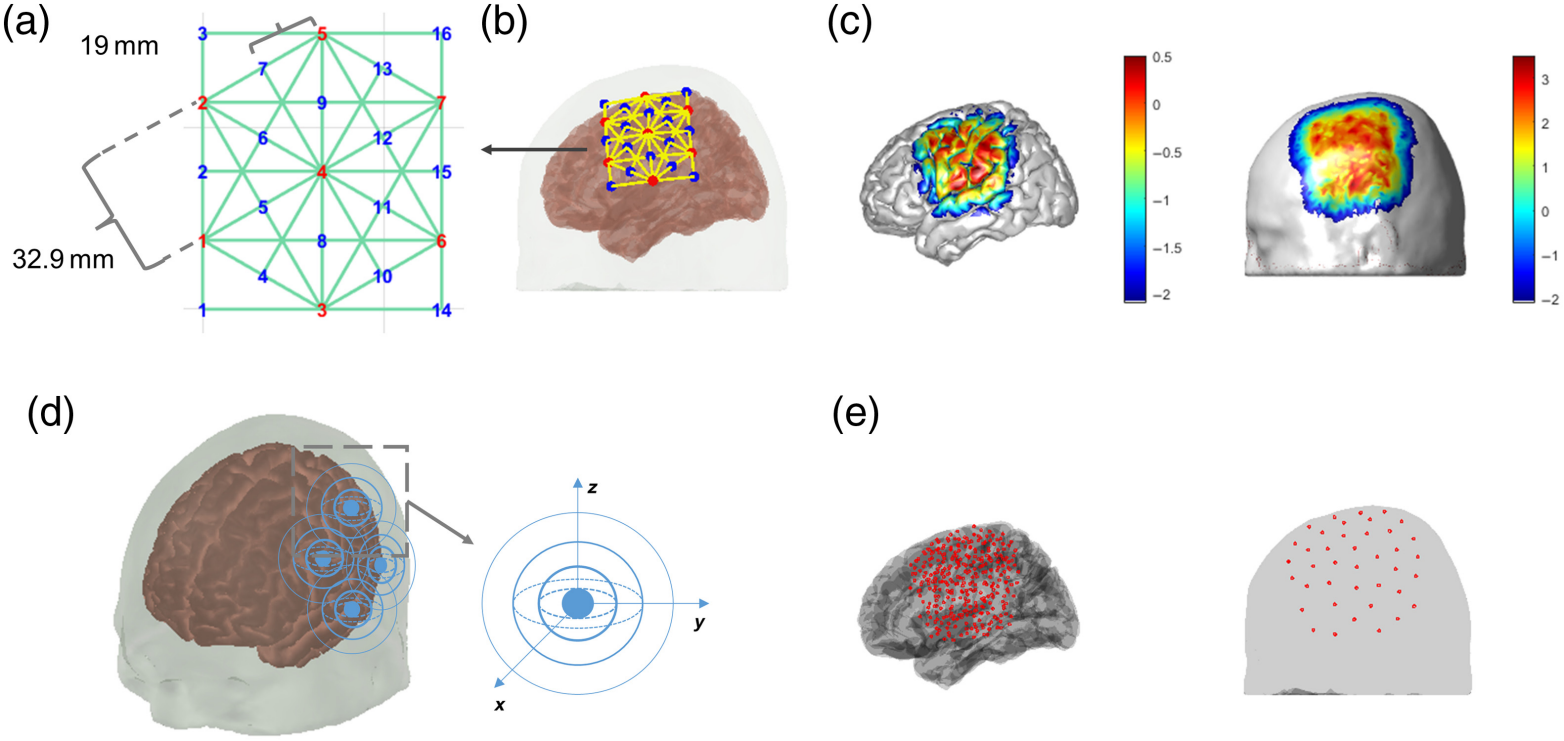
Probe design and illustration of the spatial kernels. (a) The layout of one patch of the high-density probe; red numbers are sources, and blue numbers are detectors. Green lines are channels. (b) The position of the probe patch on the head. (c) The sensitivity profile in the brain (left) and scalp (right) region on a log 10 scale. (d) The illustration of the 3D spatial Gaussian kernels. (e) The center positions of the spatial kernels of the brain (left) and the scalp (right) regions.

The high-density fNIRS probe used in this study was designed using a hexagonal pattern [Fig. 1(a)], with 7 sources and 16 detectors forming 50 channels. The SS detectors are placed 19 mm apart from the sources, and the long-separation detectors are placed 32.9 mm apart from the sources, covering a $76\times 66\;{\mathrm{mm}}^{2}$ area. This probe was placed on the motor region of the head, with only one patch on the left hemisphere in the simulation study and two patches on both hemispheres in the experimental study [Fig. 1(b)]. Following our “NinjaCap” approach, we 3D-printed the caps designed in AtlasViewer^47^ to ensure the proper positioning of the optodes on the head.^51^ We also measured the Cz position of the head to make sure the cap’s Cz marker and the head’s true Cz position were accurately aligned.

### 2.2 Spatial and Temporal Bases

As described in Ref. 49, to represent the underlying physiological changes in both the brain and scalp regions, we assumed that changes in oxy-(HbO) and deoxyhemoglobin (HbR) concentration can be described as linear combinations of a set of spatial and temporal basis functions designated to capture the functional and physiological hemodynamic fluctuations. One of the benefits of implementing the spatial basis is to reduce the degrees of freedom of the model to make the model more computationally tractable.

We employed a set of overlapping 3D Gaussian kernels (g) to represent the individual functional hemodynamic changes at each vertex of the brain and scalp surface [Fig. 1(d)]:

$$g=\exp(\frac{-{\left\|x-x_{0}\right\|}^{2}}{2\sigma_{g}^{2}}),$$ (1)

where $x_{0}$ represents the center point location of the Gaussian kernel, x represents the location of the element vertex, and $\sigma_{g}$ is the Gaussian standard deviation, which controls the width of the Gaussian kernel. The spatial bases were placed only in the regions of the head with a high-sensitivity profile (i.e., sensitivity value >0.01) to reduce computational expense. The kernels were placed 5 and 20 mm apart from each other for the cortex and the scalp, respectively [Fig. 1(e)]. The Gaussian kernel standard deviation $\sigma_{g}$ was set equal to the kernel distance. The brain and scalp spatial basis functions were restricted to the element vertices on the brain and scalp, respectively. The spatial basis sets for HbO and HbR changes in brain and scalp are denoted as $G_{\text{brain},\mathrm{HbO}}$, $G_{\text{brain},\mathrm{HbR}}$, $G_{\text{non}-\text{brain},\mathrm{HbO}}$, and $G_{\text{non}-\text{brain},\mathrm{HbR}}$, respectively.

To model the temporal dynamics of the evoked functional changes within each of these Gaussian bases, we used a linear combination of a two-parameter modified Gamma function, as given by the following equation:

$$t(t)=\frac{{\left(t-\tau \right)}^{2}}{\sigma^{2}e^{-1}}\;\exp(\frac{-{\left(t-\tau \right)}^{2}}{\sigma^{2}}),$$ (2)

where τ is the delay time of the response and σ is the parameter that controls the temporal width of the response function. This basis support is similar to the temporal basis often used in GLM analyses.^52^^,^^53^ The overall temporal model of the functional component of the hemodynamic signals is expressed as the convolution of the experimental stimulus timing (U) and the functional impulse response function:

T=t*U, (3)

where U is a binary vector describing the experimental paradigm (i.e., the timing of stimulus presentation) and spans the temporal duration of the experiment and * is the convolution operator. The dimensions of the T matrix are the number of temporal basis functions by the number of measurement time points. Here we used the same temporal basis function for the scalp region [Eq. (2)] to represent the physiological responses to the stimulus in the scalp region. The temporal basis sets for HbO and HbR changes in brain and scalp are denoted as $T_{\text{brain},\mathrm{HbO}}$, $T_{\text{brain},\mathrm{HbR}}$, $T_{\text{non}-\text{brain},\mathrm{HbO}}$, and $T_{\text{non}-\text{brain},\mathrm{HbR}}$, respectively.

Finally, all spatial–temporal bases are combined into a single Kronecker operator (GT) with a total of four unique basis groups. This matrix is formed by the Kronecker product of the individual spatial (G) and temporal (T) basis functions for each of the eight groups mentioned above. The matrix GT is given by the following equation:

$$\mathrm{GT}=[\begin{array}{cc} G_{\text{brain},\mathrm{HbO}}⊗T_{\text{brain},\mathrm{HbO}} & G_{\text{non}-\text{brain},\mathrm{HbO}}⊗T_{\mathrm{non}-\mathrm{brain},\mathrm{HbO}}\\ G_{\text{brain},\mathrm{HbR}}⊗T_{\text{brain},\mathrm{HbR}} & G_{\text{non}-\text{brain},\mathrm{HbR}}⊗T_{\text{non}-\mathrm{brain},\mathrm{HbR}} \end{array}],$$ (4)

which represents the hemoglobin changes in each vertex and each time point for each condition of stimulus. Here ⊗ represents the Kronecker product.

### 2.3 Sensitivity Matrix

We employed Monte Carlo simulations to calculate the sensitivity matrix $A_{\lambda }$ using the MCXLAB toolbox^54^ for each wavelength, in this case, for two wavelengths $\lambda_{1}$ and $\lambda_{2}$. The $A_{\lambda }$ matrix has the dimension of the number of channels by the number of voxels or vertices, and it maps the changes of the absorption coefficient values in voxels to the change in optical density values in the measurement channels. The Colin head model was used in this Monte Carlo simulation, and $10^{7}$ photons were simulated from each optode. The scattering anisotropy g does not change the result in the diffusion equation provided that the value of the reduced scattering coefficient is kept constant. The Monte Carlo simulation is more efficient for smaller values of g, so we used g=0.01.^33^ We used a refractive index of 1 for all tissues.^33^ Colin27 was used as our head model.^47^^,^^55^ The Colin head model^55^ was segmented into five types of tissues, scalp, skull, CSF, gray matter, and white matter using SPM software.^56^ The absorption coefficients $\mu_{a}$ and reduced scattering coefficients $\mu_{s}^{\prime }$ used for simulations are listed in Table 1.^57^^–^^60^

**Table 1:** Absorption coefficients $\mu_{a}$ and reduced scattering coefficients $\mu_{s}^{\prime }$ for different tissue layers.

Tissues	$\mu_{a}$ (${\mathrm{mm}}^{-1}$)		$\mu_{s}^{\prime }$ (${\mathrm{mm}}^{-1}$)	
	760 nm	850 nm	760 nm	850 nm
Scalp	0.0177	0.0190	0.73	0.64
Skull	0.0125	0.0139	0.93	0.84
CSF	0.0021	0.0040	0.30	0.30
Gray matter	0.0195	0.0192	1.18	0.67
White matter	0.0195	0.0208	1.18	1.01

The foby

$$A=(\begin{array}{cc} \epsilon_{\lambda_{1}}^{HbO}A_{\lambda_{1}} & \epsilon_{\lambda_{1}}^{HbR}A_{\lambda_{1}}\\ \epsilon_{\lambda_{2}}^{HbO}A_{\lambda_{2}} & \epsilon_{\lambda_{2}}^{HbR}A_{\lambda_{2}} \end{array}),$$ (5)

where ϵ is the extinction coefficient for the chromophores (HbO and HbR) at each wavelength $\lambda_{i}$.^61^

### 2.4 Forward Model

We first converted the light intensity into the changes in optical density values by

$$\Delta \mathrm{OD}(t)=-\log(\phi (t)/\phi_{0}),$$ (6)

where ΔOD is the change in the optical density, the logarithm is a common log, and $\phi_{0}$ is the average detected photon fluence. Before the conversion, we prune the noisy channels with a signal-to-noise ratio (SNR) lower than 5 using the hmrR_PruneChannels function in Homer3. The SNR is defined as the mean divided by the standard deviation of the signal. After the conversion, motion artifacts are corrected by the SplineSG method using the hmrR_MotionCorrectSplineSG function in Homer3, with the interpolation parameter set to 0.99.^62^

The measurement from the channels was modeled as a linear combination of four components: (1) stimulus-derived cerebral activity, (2) stimulus-derived extracerebral activity, (3) global systemic physiology, and (4) drift in the measurement itself. The first two parts are described as a linear sum of the spatial–temporal basis function GT weighted by a vector of unknown coefficients denoted by $b_{f}$:

$$[\begin{array}{c} \Delta \mathrm{HbO}\\ \Delta \mathrm{HbR} \end{array}]=\mathrm{GT}\cdot b_{f}.$$ (7)

The optical density changes are obtained by

$$\Delta {\mathrm{OD}}_{f}=A\cdot \mathrm{GT}\cdot b_{f},$$ (8)

where the subscript f represents the functional changes.

Global systemic physiology is captured by the measurement in the SS channels denoted by $\Delta {\mathrm{OD}}_{\mathrm{SS}}$, and the signal drift is represented by drift regressors denoted by ${\mathrm{OD}}_{\text{drift}}$. We denote the measurement from channels in the high-density probe (including the long 32.9 mm channels and the shorter 19 mm channels) as ΔOD. Consequently, the forward model is written as

$$\Delta \mathrm{OD}=[\Delta {\mathrm{OD}}_{\mathrm{SS}},{\mathrm{OD}}_{\text{drift}},A\cdot GT]\cdot b,$$ (9)

where b are the unknown coefficients for SS regressors, drift regressors, and the functional changes.

This final model is written as a single matrix equation:

$$\Delta \mathrm{OD}=[\Delta {\mathrm{OD}}_{\mathrm{SS}},{\mathrm{OD}}_{\text{drift}},H]\cdot b,$$

Y=O·b, (10)

where Y is the concatenated set of measurement channels for all time points, i.e., ΔOD; O is $\left[\Delta {\mathrm{OD}}_{\mathrm{SS}},{\mathrm{OD}}_{\text{drift}},H\right]$; and H is A·GT.

### 2.5 Inverse Model and Regularization

First, we employ spatial regularization,^9^ which is realized by replacing A in Eq. (9) by A^. A^ is the A matrix spatially regularized by the diagonal matrix L:

A^=AL−1, (11)

$$\mathrm{diag}(L)=\sqrt[{2}]{\mathrm{diag}(A^{T}A)+\eta }.$$ (12)

To estimate the coefficients of the spatial–temporal basis sets, the linear model Eq. (10) is solved using the Tikhonov regularization scheme (Moore–Penrose generalized inverse), with spatial regularization:^9^

$$b=L^{-1}(O^{T}Y)/(O^{T}O+\alpha I_{f}),$$ (13)

where α is the regularization parameter and $I_{f}$ is a diagonal matrix that has diagonal values of ones for the regressors of A·GT and zeros for the regressors of $\Delta {\mathrm{OD}}_{\mathrm{SS}}$ and ${\mathrm{OD}}_{\text{drift}}$ as we only regularized the functional changes to balance the high spatial frequency noise that would otherwise appear in the image because of the ill-conditioned A·GT matrix. The Tikhonov parameter α adjusts the balance between high spatial frequency noise and image smoothness and was set to 0.01 times the maximum diagonal value of $O^{T}O$. The spatially variant regularization parameter η was set to 0.01 times the maximum diagonal value of A^TA^.^60^^,^^63^ The GPU acceleration method for this model is presented in the Supplementary Material.

### 2.6 Sequential Models

The sequential model refers to a model that first performs SS regression in the channel-wise space and then carries out the image reconstruction.^8^ To do the regression of SS in the channel-wise space, we use the modified Beer–Lambert law to convert the measured OD time series data from each channel to Hb signals:

$$(\begin{array}{c} {\Delta \mathrm{OD}}_{\lambda_{1}}\\ {\Delta \mathrm{OD}}_{\lambda_{2}} \end{array})=(\begin{array}{cc} \epsilon_{\lambda_{1}}^{\mathrm{HbR}}\mathrm{ppf} & \epsilon_{\lambda_{1}}^{\mathrm{HbO}}\mathrm{ppf}\\ \epsilon_{\lambda_{2}}^{\mathrm{HbR}}\mathrm{ppf} & \epsilon_{\lambda_{2}}^{\mathrm{HbO}}\mathrm{ppf} \end{array})(\begin{array}{c} \Delta \mathrm{HbR}\\ \Delta \mathrm{HbO} \end{array}),$$ (14)

where ϵ is the extinction coefficient and ppf is the partial pathlength factor.^64^^,^^65^

Then we construct the GLM on the Hb signals:

$$\Delta \mathrm{Hb}=[\begin{array}{ccc} T & \mathrm{SS} & D \end{array}]\cdot \beta +\varepsilon ,$$ (15)

where T is the convolution of modified gamma function and the stimulus sequence as in Eq. (3), SS is the short-separation regressor, D is the drift regressor matrix, β is the unknown coefficients assigned to each regressor, and ε is the residual term. Here β are the model coefficients to be estimated and can be approximated as β^ by solving the inverse problem:

$$J=[\begin{array}{ccc} T & \mathrm{SS} & D \end{array}],$$ (16)

β^=(JTJ)−1JTΔHb. (17)

Then we derive the HRF function:

HRF=t·β^′, (18)

where β^′ is a subset of β^ that only contains the coefficients assigned to the regressors T. t is the modified gamma function defined in Eq. (2). We convert the HRF to ΔOD using the modified Beer–Lambert law [Eq. (14)]. The ΔOD is the input to the image reconstruction step:

$$(\begin{array}{c} {\Delta \mathrm{OD}}_{\lambda_{1}}\\ {\Delta \mathrm{OD}}_{\lambda_{2}} \end{array})=A(\begin{array}{c} \Delta \mathrm{HbO}\\ \Delta \mathrm{HbR} \end{array}),$$ (19)

where A is the sensitivity matrix in Eq. (5) that we derived from Monte Carlo simulation (see Sec. 2.3). ΔHbO and ΔHbR, the two unknowns in this model, are the Hb values at each vertex in the 3D head model. Equation (19) is rewritten as

y=A·b, (20)

which is solved as an inverse problem:

$$b=A^{-1}\cdot y,$$ (21)

where $A^{-1}$ is the Moore–Penrose pseudoinverse with Tikhonov regularization:

A−1=L−1(A^TA^+λ1I)−1A^T, (22)

where A^ is the A matrix spatially regularized by the term L, as defined in Eqs. (11) and (12). The Tikhonov parameter $\lambda_{1}$ adjusts the balance between high spatial frequency noise and image smoothness and was set to 0.01 times the maximum diagonal value of $A^{T}A$.^60^^,^^63^

Three SS conditions were tested: (i) no SS regression; (ii) average SS; and (iii) fake SS. The descriptions of (ii) and (iii) are in Sec. 2.4.

Two image reconstruction models were tested: (i) head: $A=[A_{\text{brain}}\;A_{\text{non}-\text{brain}}]$, where $A_{\text{brain}}$ contains vertices only from brain cortex and $A_{\mathrm{non}-\mathrm{brain}}$ has all vertices that are not within the brain cortex, and (ii) brain only: $A=[A_{\text{brain}}]$.

### 2.7 Model Performance Evaluation Metrics

The images are created using the values at the time-to-peak. Time-to-peak is always the same time point as we used a predefined modified gamma function to model the HRF curve. We used two metrics to evaluate the performance of each model: the CBR and t-value. Both metrics are calculated at the time point of the highest HRF amplitude. We calculated CBR for the image of each subject as

$$\mathrm{CBR}=\frac{\text{mean}(V\cdot I_{\mathrm{ROI}})}{\mathrm{std}(V\cdot I_{\text{background}})},$$ (24)

where V is the Hb values in one image, $I_{\mathrm{ROI}}$ is the mask array of the vertices that are within the region of interest (ROI), and $I_{\text{background}}$ is the mask array of the vertices that are within the background region. After calculating the CBR value for each image on the subject level, we averaged it over all subjects to derive the mean CBR value as the second evaluation matrix.

The t-value is calculated as

$$t-\text{value}=\frac{\overset{¯}{V}}{\sigma /\sqrt{n}},$$ (23)

where $\overset{¯}{V}$ represents the mean Hb value within the ROI/Hb value within the background region averaged across the subjects, σ represents the standard deviation of mean Hb value within the ROI/background region across the subjects, and n represents the number of subjects. To define the ROI region and the background region, we input a simulated signal that contains only synthetic HRF (no resting data) into the SS-DOT model and derived a noise-free HbO image. In this image, if the Hb value of a vertex was above 90% of the maximum value, it was defined as within the ROI; if the Hb value of a vertex was below 1% of the maximum value, it was defined as within the background region. The ROI region and the background region are shown in Fig. 4(e).

For the experimental dataset, we further calculated a laterality index (LI):^66^^,^^67^

LI=(C−I)/(I+C), (25)

where I represents the number of vertices that have Hb values larger than half of the maximum activation on the ipsilateral hemisphere and C refers to the same value on the contralateral hemisphere. LI was calculated for each subject’s reconstructed image as well as for the group average image. The subject level LI was compared using the Kruskal–Wallis test between the models at the significance level of 0.05. The Kruskal–Wallis test was used instead of one-way ANOVA because the LI data are not normally distributed. The *post hoc* multiple comparison was corrected by the Bonferroni correction method.^68^

### 2.8 Computational Load

The simultaneous model is computationally consuming. The runtime of processing the experimental dataset (12 subjects, 3 runs per subject, and 8 mins recordings per run) on a CPU (Intel i9-9900K) is around 40 s for the sequential models, whereas it is about 62 min for the simultaneous models. We developed a GPU algorithm (described in the Supplementary Material) to accelerate it from 62 to 28 min on NVDIA GeForce GTX 1660 Ti.

## 3 Experiments

### 3.1 Simulation Study Design

To test the performance of the model, we simulated datasets by adding synthetic HRF to experimental resting-state data from 11 subjects (see Sec. 3.2). The model was thoroughly examined under various synthetic HRF amplitudes.

**Fig. 2:**
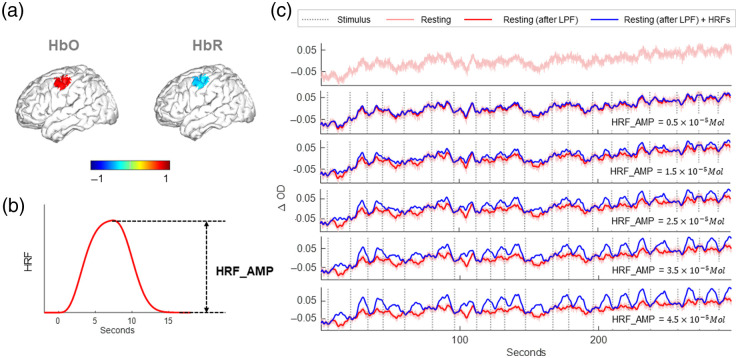
Simulation of fNIRS data. (a) The perturbation location of both HbO and HbR. The color bar is normalized by the maximum HbO value. (b) The simulated HRF shape. The height of the HRF is represented by HRF_AMP. (c) An example of resting data and resting data overlayed with HRF sequences with three different HRF amplitudes. LPF, low-pass filter of 0.5 Hz.

To form the synthetic HRF, we simulated a perturbation of HbO and HbR in the image space in the motor region on the brain cortex as a step function in the space with a blob diameter of 10 mm [see Fig. 2(a)]. The amplitude of HbR was half of HbO but negative. In the temporal domain, the brain response time series data are the convolution of the modified gamma function in Eq. (2) [the shape is presented in Fig. 2(b)] and the stimulus sequence (convoluted HRF, abbreviated as cHRF). The stimulus sequences are the same as the sequences used in experimental design but unified as one stimulus condition. Three replications were carried out with different stim marker sequences with randomized ISI. Then we employed the forward matrix A in Eq. (5) to project the perturbation from image space to channel space. In Fig. 2(c), we show an example of the experimental resting state data and the data with cHRF overlayed onto it. We simulated five different HRF amplitudes, from $0.5\times 10^{-5}$ to $4.5\times 10^{-5}\;M$ with linear steps of $1\times 10^{-5}\;M$.

We tested two different models of SS regressors: (i) average SS: the average OD data over all 19 mm channel time series after first adding the simulated HRF to each channel (see Sec. 3.1) and (ii) fake SS: the average OD data over all 19 mm channels without adding the simulated HRF. The method (ii) is called fake SS because there was no physical 8 mm channel on the probe.

### 3.2 Experimental Study Design

**Fig. 3:**
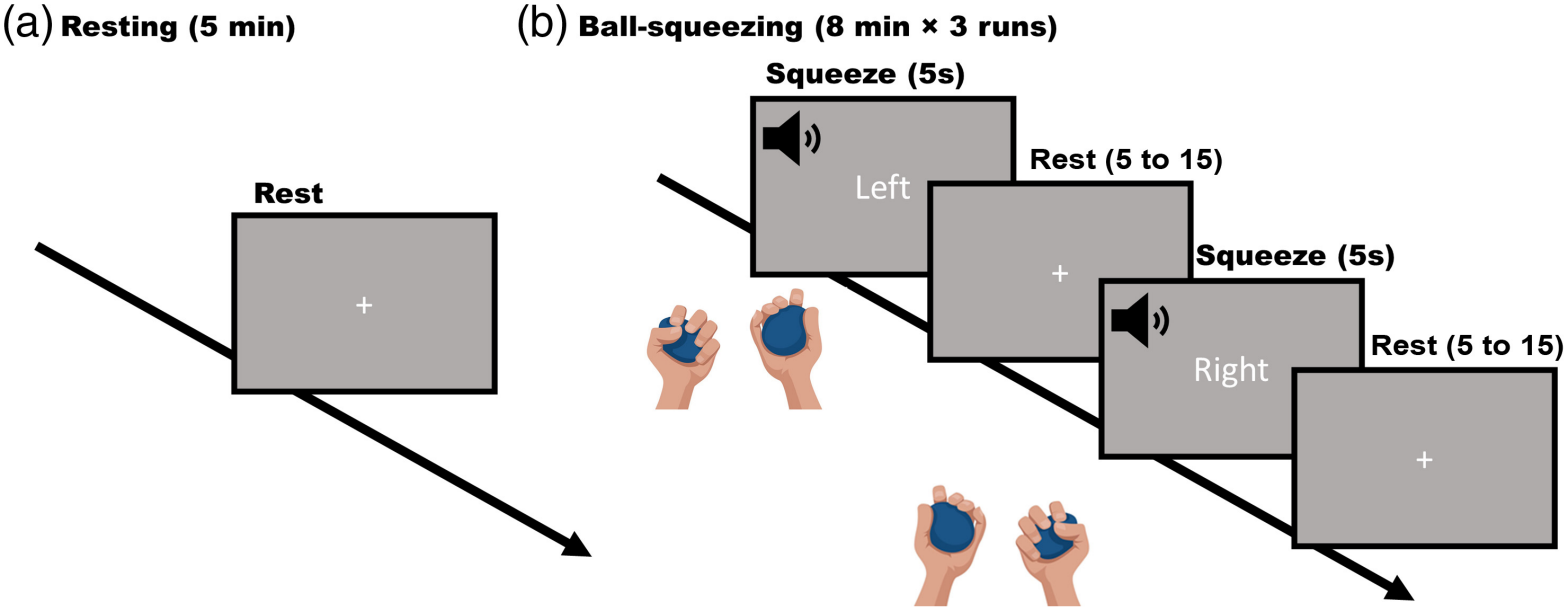
Experimental paradigm: (a) the 5-min resting state task and (b) the ball-squeezing task.

Study protocol was approved by the Institutional Review Board of Boston University. All participants provided written informed consent to take part in this study. All participants were healthy young adults (n=12). 11 participants went through one run of 5-min resting state data collection, and all 12 participants went through three 8-min runs of left- and right-hand ball-squeezing task (illustrated in Fig. 3). During the resting state measurement, we asked participants not to move and to remain mentally idle as much as possible for 5 min. During the ball-squeezing task, the participants sat in front of a computer screen (13.3 in. with resolution of 2560×1600) and held one rubber ball (2.5 in. in diameter) in each hand. When the task started, they squeezed the ball in either the left or the right hand according to a visual cue shown on the computer screen accompanied by an audio sound. The stimulus was 5 s, and the rest in between was randomly varied between 5 and 15 s. In each run, there were 15 trials with left-hand stimulus and 15 trials with right-hand stimulus in a fully randomized order. The stimulus presentation was created with PsychoPy software.^69^ fNIRS signals were measured throughout the experiment (two cascaded NIRSport2 16×16 devices, NIRx, Berlin, Germany). The probe design was the same layout as in the simulation design [Fig. 1(a)], with two patches covering the motor regions on each hemisphere.

## 4 Results

### 4.1 Simulation Results

**Fig. 4:**
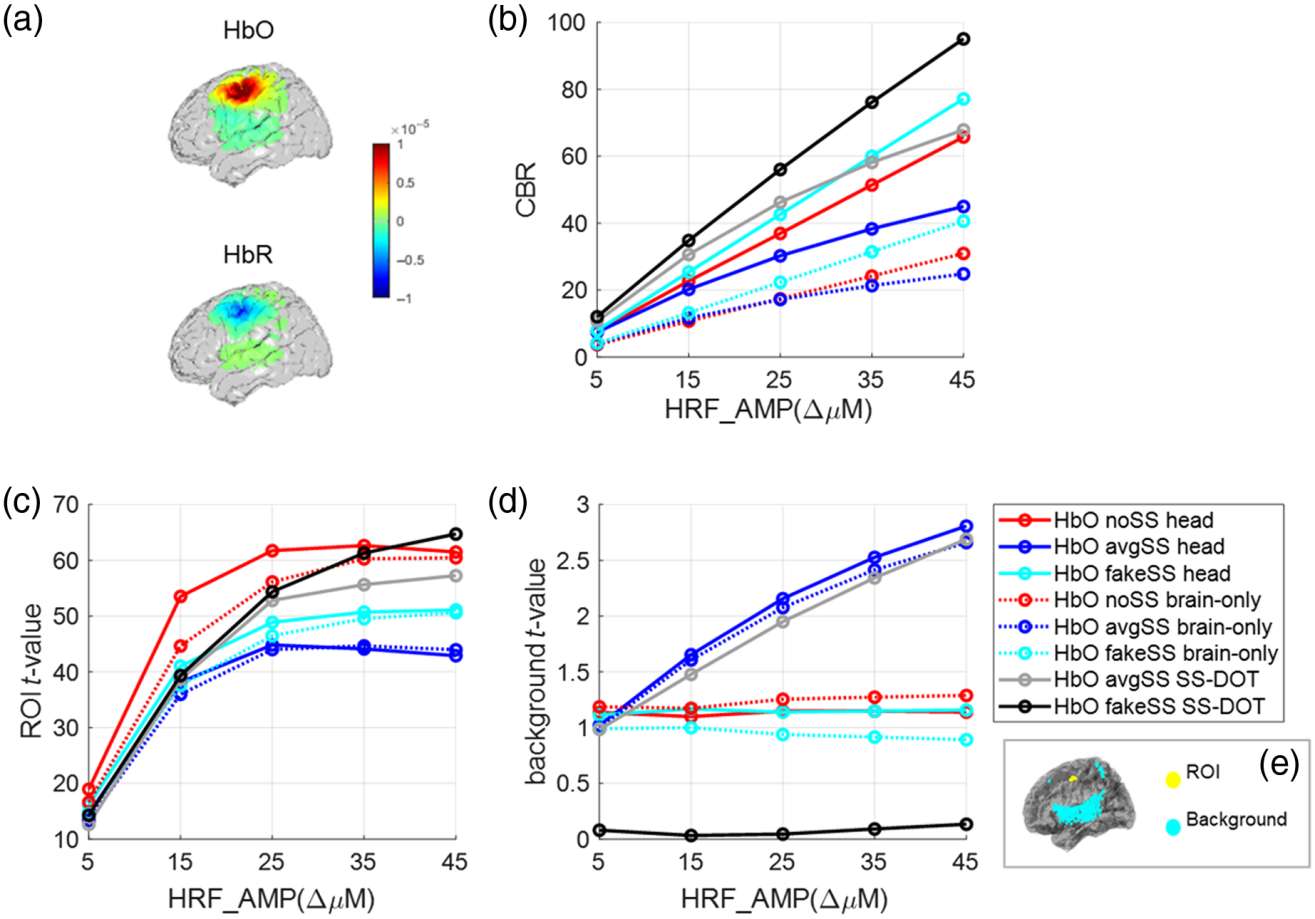
Results from the simulated datasets (HbO). (a) An example of the image reconstructed by the fakeSS SS-DOT model (subject #12, HRF amplitude=25  μM). (b) The CBR value under different HRF amplitudes for each model. The average t-value in the (c) ROI region and (d) background region under different HRF amplitudes for each model. (e) The ROI region and the background region.

The CBR and t-values at different HRF amplitudes are shown in Fig. 4 for HbO images (see Fig. S2 in the Supplementary Material for HbR) for eight different models: six sequential models, “noSS head,” “avgSS head,” “fakeSS head,” “noSS brain only,” “avgSS brain only,” and “fakeSS brain only,” and two simultaneous models, “avgSS SS-DOT,” “fakeSS SS-DOT.” With increasing simulated HRF amplitudes, CBR shows an increasing trend for all models [Fig. 4(b)]. The fakeSS SS-DOT provides the highest CBR value compared with the other models. However, when the HRF amplitude is small, the improvement is marginal. For sequential models, out of the three SS methods, the “fakeSS” model has the greatest CBR. Out of the two DOT methods, i.e., head and brain-only, whole head reconstruction has higher CBR than brain-only reconstruction. The observation holds true for the HbR CBR values except that noSS head has a higher CBR than fakeSS head with a negligible margin (see Fig. S2B in the Supplementary Material). The error bar is not shown in Fig. 4(b) or Fig. S2B in the Supplementary Material because it would make the visualization difficult. The plots with the error bars can be found in Fig. S9 in the Supplementary Material. The inferential statistics are shown in Tables S7 and S8 in the Supplementary Material.

The t-value in the ROI region [Fig. 4(c)] has an increasing trend with increasing HRF amplitude, whereas the avgSS models decrease at larger HRF amplitudes. The noSS models show the highest t-value except that the fakeSS SS-DOT model exceeds at the largest HRF value. Comparing the two DOT models, the head models always show a higher t-value than brain-only models.

The t-value in background region [Fig. 4(d)] increases for avgSS models, but it is not affected with increasing HRF amplitude for other models. The fakeSS SS-DOT model has the lowest background t-value for HbO but not for HbR (Figs. S2C and S2D in the Supplementary Material).

### 4.2 Experimental Results

**Fig. 5:**
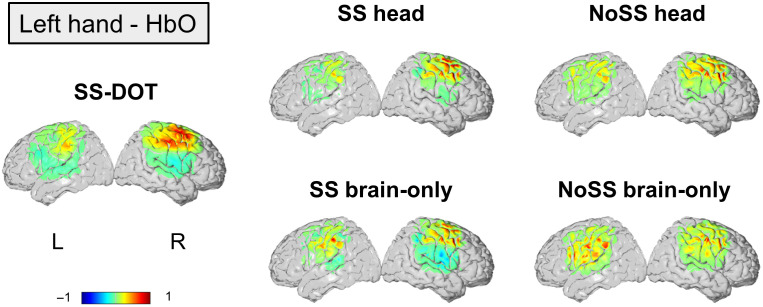
Group average HbO images for each model under the condition of left hand. The color bar is normalized by the maximum HbO value.

**Fig. 6:**
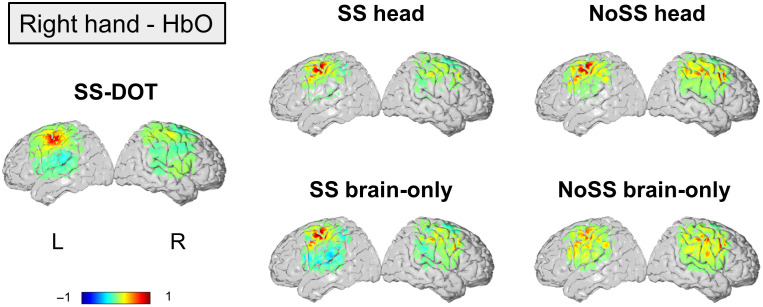
Group averaged HbO images for each model under the condition of right hand. The color bar is normalized by the maximum HbO value.

**Fig. 7:**
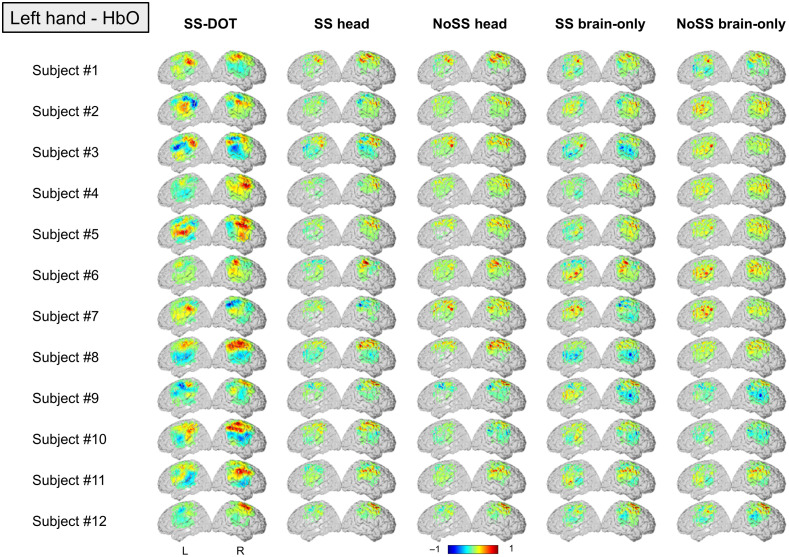
Subject level HbO images for each model under the condition of left hand. The color bar is normalized by the maximum HbO value.

**Fig. 8:**
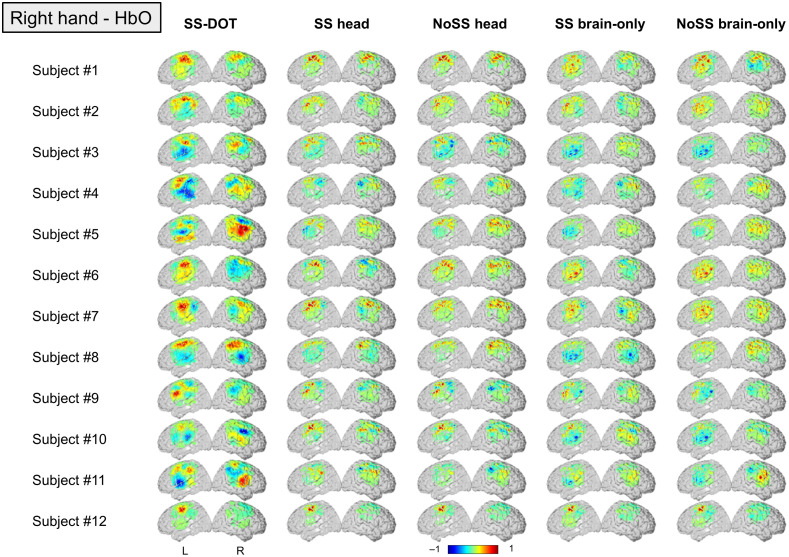
Subject level HbO images for each model under the condition of right hand. The color bar is normalized by the maximum HbO value.

In Figs. 5–8, the group average and individual reconstructed HbO images are presented for the 12 subjects that performed the ball squeezing task. A higher HbO activation is expected on the contralateral hemisphere to the hand performing the ball squeezing task.^70^ In Figs. 5 and 6, we observe such trends in the group level results for all models, except for “No SS brain-only” under the left-hand condition. The LI values for group average images (Table 2) also support that only NoSS brain-only model has a negative LI value.

**Table 2:** t-value from subject level images, CBR, and LI value derived from group averaged image for each model on experimental data (HbO). The bold values are the greatest among the models.

HbO		SS-DOT	SS head	noSS head	SS brain-only	noSS brain-only
Left hand	t-value	2.63	2.60	**3.21**	2.60	3.05
	CBR	**3.10**	1.42	1.03	0.55	0.45
	LI	**1.00**	**1.00**	**1.00**	0.71	−0.20
Right hand	t-value	3.00	2.71	**3.35**	2.64	3.14
	CBR	6.55	**0.87**	0.62	0.52	0.39
	LI	**1.00**	**1.00**	0.42	0.72	0.14

**Fig. 9:**
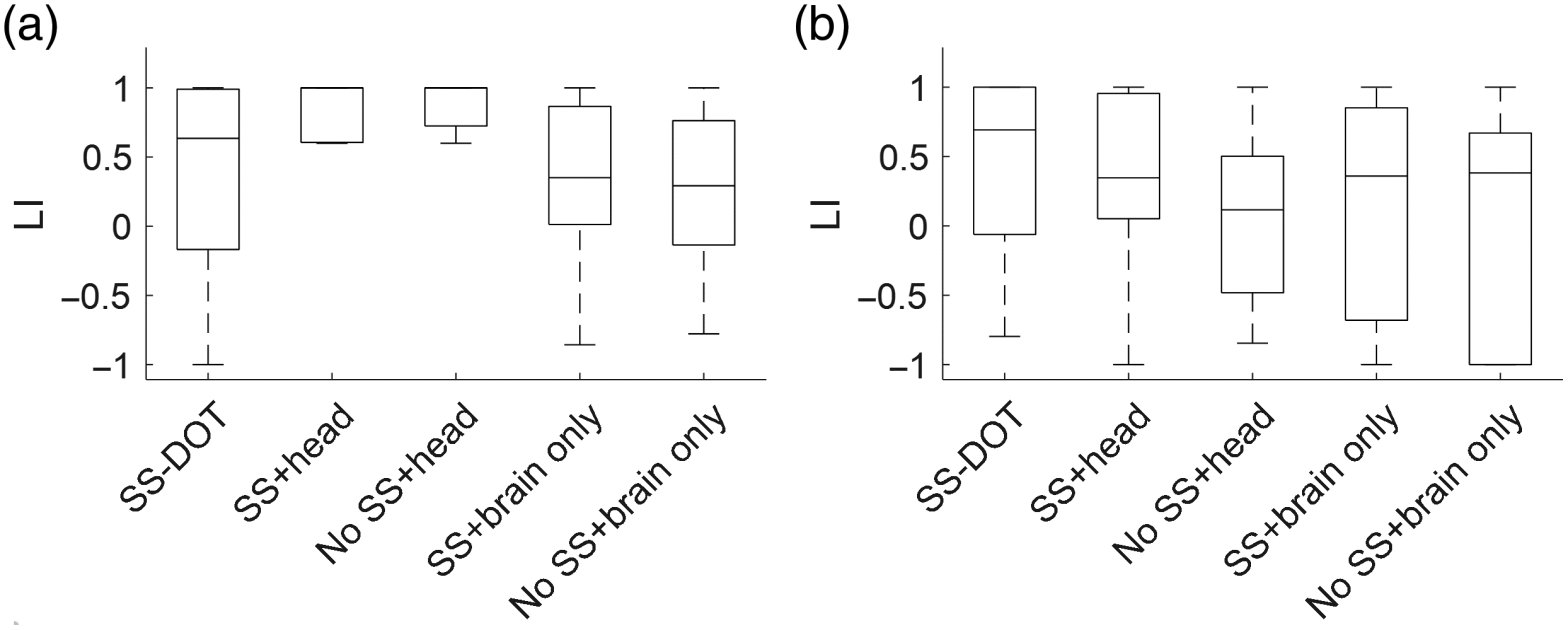
LI of HbO for each model on the experimental dataset for (a) left-hand condition and (b) right-hand condition.

In the subject-level results, the contralateral hemisphere activation is not obvious for the two brain only models (Figs. 7 and 8). For the noSS head model, the left-hand condition shows such trends but not the right-hand condition. The LI values of the subject-level images (Fig. 9) also indicate the same: the two brain only models have lower LI values (not statistically significant, inferential statistics are shown in Table S3–S6 in the Supplementary Material). Of the four sequential models, the SS head model shows the best results as both the group level and the subject level results show contralateral activation and higher LI values (not statistically significant, inferential statistics are shown in Tables S3–S6 in the Supplementary Material).

Next, we compare the best sequential model (i.e., SS head model) with the SS-DOT model. The results from SS-DOT are consistent with what we expected both at the group-level and the subject-level. Under the left-hand condition, the laterality is more obvious both on the group level and the subject level compared with “SS head,” but with a larger activation area. The LI values for the SS-DOT model were not significantly different from the SS head model.

The HbR results are also shown in Figs. S3–S6 in the Supplementary Material. The subject-level HbR images were noisier than HbO images, with only group level images showing a meaningful trend. Consistent with the HbO results, the SS head model outperformed the other sequential models. For the SS-DOT model, the group level results also showed lower HbR on the contralateral hemisphere. The LI values were not statistically significantly different across the models (Fig. S7 in the Supplementary Material).

We also calculated CBR and t-values for the experimental images (HbO results are shown in Table 2). The SS-DOT model shows higher CBR values than the sequential models, and the ‘NoSS head’ models show the highest t-values, which is consistent with the simulation results. This can be explained by visually inspecting Figs. 7 and 8, which show that the variance of the images across the subjects is large. A similar trend can also be observed in HbR values, which are presented in Table S2 in the Supplementary Material for the completeness of the results.

**Fig. 10:**
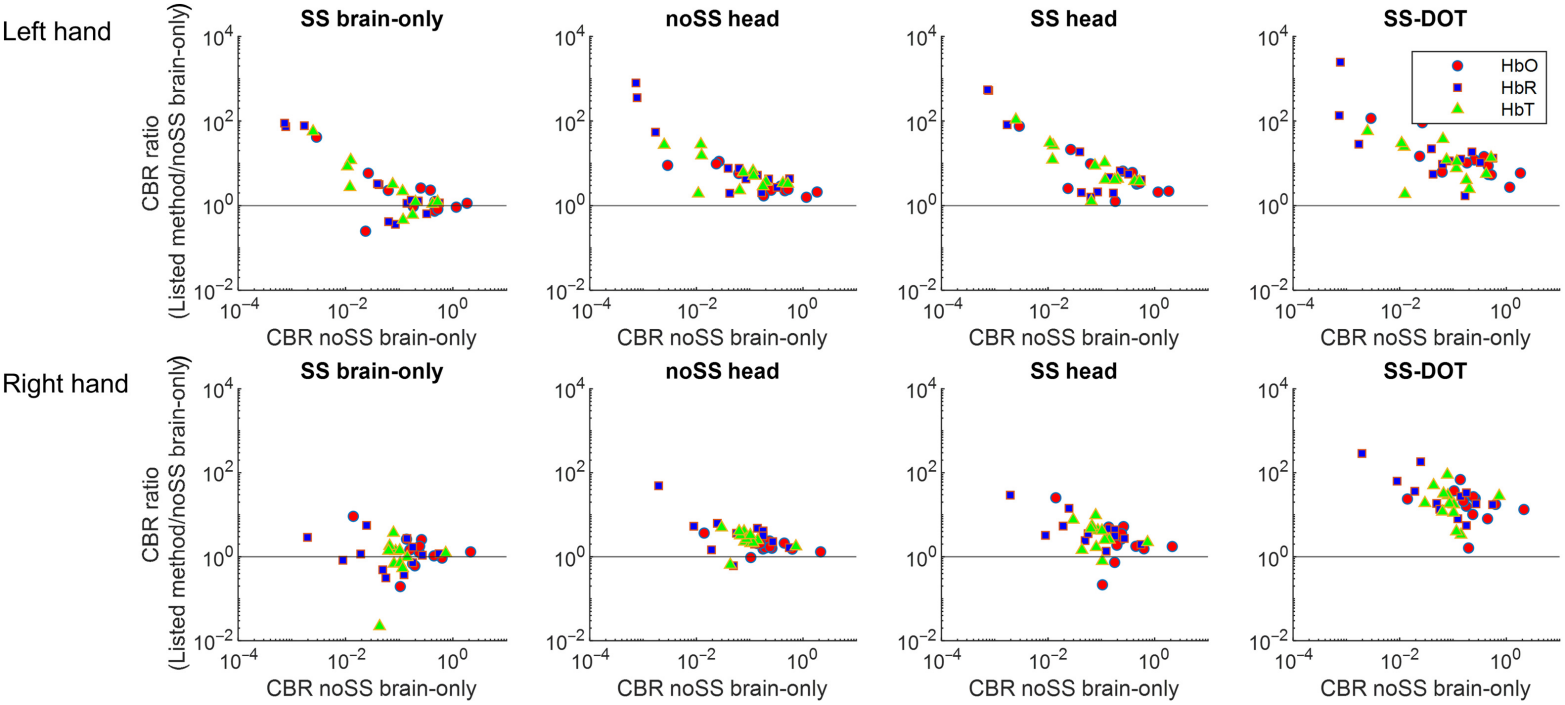
Evaluation of CBR for the experimental dataset (12 subjects). All graphs plot the ratio of improvement in SBR versus the model noSS brain-only. HbO, HbR, and HbT are denoted in red circles, blue squares, and green triangles, respectively.

The ratio of improvement in CBR versus noSS brain-only for each subject is shown in Fig. 10. With either SS and/or whole head DOT added to the model, the CBR values increase, especially for the subjects who have lower CBR when neither SS nor whole head image reconstruction is implemented. With SS and whole head DOT added to the sequentially model, the CBR is further improved compared with only one of them being implemented. Based on this, the CBR further increases with the SS-DOT model in which SS and whole head DOT are simultaneously implemented.

## 5 Discussion

In this work, we propose a model, SS-DOT, which performs SS regression and DOT image reconstruction methods simultaneously. To test the performance of the new model, we designed a high-density fNIRS probe, and using this probe, we measured fNIRS data from 12 healthy subjects while they were performing a resting-state task and a ball-squeezing task. Based on the resting-state data that we collected, we simulated an fNIRS dataset by overlaying the resting-state data with a synthetic HRF. We also tested two different SS separation models: avgSS uses the average of all channels shorter than 19 mm as the SS regressor after overlaying it with synthetic HRF and fakeSS uses the same averaged signal before the overlay. The avgSS method was motivated by Ref. 8. The fake SS was innovated because we did not have ∼8  mm SS channels in our probe due to physical limitations. On both simulated and experimental ball-squeezing datasets, we thoroughly tested the SS-DOT model and other sequential models quantitively and qualitatively. From the simulation results, fakeSS SS-DOT showed greater CBR values but only showed marginal benefits at small HRF amplitudes. From the experimental ball-squeezing study, the results are consistent with the simulation study as SS-DOT has the greatest CBR value. The LI values are not significantly different among the models. This indicates the potential of the SS-DOT model to improve the image quality of fNIRS data reconstruction, especially for larger HRF amplitudes. The conventional sequential models are still suitable and practical enough in most cases as they show ability to separate the brain activation from the extracerebral physiological noise in this study.

It was demonstrated that performing SS regression before the DOT image reconstruction could result in an increased contrast-to-noise ratio and decreased image variance across blocks.^8^ We hypothesized that performing SS regression and DOT simultaneously would perform better than performing each separately. Our results show that the SS-DOT model improves the image quality by increasing the CBR value. However, the benefits are marginal at small HRF amplitudes, in which case, the sequential model performs as good as the simultaneous model. This work also presents the performance of other sequential models, thus contributing the information of which SS method and which DOT method would perform better. The no SS model shows the highest t-value, followed by the fakeSS and avgSS models. We speculate that the SS regressor, whether it is 8 mm measurement or average of 19 mm measurements, is prone to removing the brain activation signal, thus lowering the statistical power to detect the brain activation. The whole head DOT models always outperform the brain-only DOT models. Of note, the t-value would be affected by different sample sizes, so one should be careful when comparing our t-values with other studies that have different sample sizes.

Our simulation results showed that CBR goes down for the avgSS models but not for the other models. This is because, when the HRF amplitude increases, the average of the 19 mm SS channels contains more brain activation signals (Fig. S8 in the Supplementary Material). Thus regressing it out results in removing the brain signals. With smaller HRF amplitudes, regressing it also removes part of the brain signals; however, because the brain signal is relatively small compared with large HRF amplitude, it does not affect CBR values as much. Second, we also acquired the experimental fNIRS dataset using ball squeezing task to examine the performance of the sequential and simultaneous models. The results from the sequential models are comparable to Gregg et al.’s work.^8^ However, there are differences in methodologies between the two studies. For example, SS was not regressed out within a GLM framework but rather was subtracted from the long channel signal using the correlation coefficient between the SS and long channel signals as weight. Moreover, HRF was obtained using block averaging. Thus one of the evaluation metrics contrast-to-noise ratio, which was defined as peak response over standard deviation in the prestimulus baseline, could not be employed in this work. This is because our models only result in the modified gamma function, which has no variation in the pre-stimulus baseline. Despite these differences, performing SS regression and then applying DOT has the best performance among the sequential methods, supporting the findings from Gregg et al.^8^

Because we did not have ∼8  mm SS channels in our probe due to physical limitations, we simulated a “fake” SS channel in our simulation dataset by averaging the resting state data over all of the shorter channels (i.e., 19 mm channels). Because it was only resting state data without overlaying any HRFs, the signal should have not contained any stimulus driven brain activation signals. Thus regressing it out should not present a risk of removing any brain signals, which is supported by the results in Fig. 4(b). However, there is a concern that the non-brain region could also be activated physiologically by the stimulus, which was not presented in this fake channel. Thus this fake channel does not perfectly simulate the real SS data. In the future, with miniaturized optode designs, SS detectors could be implemented in high-density probes, which will allow us to investigate this further.

Future directions include the regularization methods and full-width at half-maximum analysis. The conventional Tikhonov regularization with varying depth sensitivity method was adopted. However, exploratory future research will focus on whether the added information in the SS-DOT model in the matrix before inversion changes singular value spectrum and different regularization parameters or regularization methods would yield better results.

Solving the SS-DOT model, especially the matrix multiplication step, is computationally demanding on a CPU and thus not practical in real-world settings. We took advantage of the GPU to reduce the whole processing time. Despite the GPU acceleration, the job can become even more demanding with more stimulation conditions, number of channels, longer time of measurement, and larger brain coverage. However, we foresee that, in the future, the fast development of GPU hardware and algorithms will resolve this issue.

## 6 Conclusions

The SS-DOT model improves the image quality by increasing the CBR values by threefold compared with sequential models. However, the improvement is marginal when the HRF amplitude is small. The sequential model still has a strong ability to separate the brain signal from the extracerebral physiological noises if performed with SS and whole head DOT.

## Data Availability

The code and data associated with this manuscript will be offered on the bfNIRS cloud service^71^ after acceptance.
