# Supplementary material for: Short-separation regression incorporated diffuse optical tomography image reconstruction modeling for high-density functional near-infrared spectroscopy
Source: Neurophotonics. 2023 May 23;10(2):025007. doi: 10.1117/1.NPh.10.2.025007 (PMC10203730; doi:10.1117/1.NPh.10.2.025007)
Supplement: Supplementary file 1 [file NPh_010_025007_SD001.pdf]

Supplementary information

GPU acceleration

The matrix $\boldsymbol{GT}$ has dimensions of the number of vertices multiplied by the number of spatial kernels ($\boldsymbol{G}$) by the number of discrete measurement time points by the number of temporal kernels by the number of stimulus types ($\boldsymbol{T}$) by the number of chromophore types. For our probe design in Fig. 1A, the dimensions of each term are listed in Table S1.

Table S1. The dimensions of the matrices. The values shown are from example data in simulation study and experimental study. The values varied in different data instances.

Syntax	notation	Simulation study (brain;extracerebral)	Experimental study (brain; extracerebral)
Number of vertices	$n_{vertices}$	1576; 1273	3277; 2658
Number of spatial kernels	n_g	376; 39	843; 101
Number of time points	n_{time_points}	2578	4293
Number of temporal bases	n_t	1	1
Number of stimulus condition	n_s	1	2
Number of chromophore types	n_{Conc}	2	2
Number of LS channels	$n_{channels}$	50	100
Number of SS channels	n_{SS}	1	1
Number of measurement points (ΔOD_{LS})	$n_{measurement} = n_{time\_points} \times n_{channels} \times n_{wavelength}$	$2578 \times 50 \times 2 = 257800$	$4293 \times 100 \times 2 = 858600$
Dimension of short separator regressors ($\Delta \boldsymbol{OD_{SS}}$)	$[n_{measurement}, n_{channels} \times n_{wavelength} \times n_{SS}]$	$[257800, 50 \times 2 \times 1]$	$[858600, 100 \times 2 \times 1]$
Order of drifters	n_{order}	3	3
Dimension of drifters regressors ($\boldsymbol{OD_{drift}}$)	$[n_{measurement}, n_{channels} \times n_{wavelength} \times (n_{order} + 1)]$	$[257800, 50 \times 2 \times 4]$	$858600, 100 \times 2 \times 4$
Dimension of matrix $\boldsymbol{A}$	$[n_{channels} \times n_{wavelength}, n_{vertices} \times n_{conc}]$	$[50 \times 2, 1576 \times 2]$; $[50 \times 2, 1273 \times 2]$	$[100 \times 2, 3277 \times 2]$; $[100 \times 2, 2658 \times 2]$
Dimension of matrix $\boldsymbol{GT}$	$[n_{vertices} \times n_{conc}, n_{time\_points}, n_t \times n_s \times n_g]$	$[1576 \times 2,2578,1 \times 1 \times 376]$; $[1273 \times 2,2578,1 \times 1 \times 39]$	$[3277 \times 2,4293,1 \times 1 \times 843]$; $[2658 \times 2,4293,1 \times 1 \times 101]$
Dimension of matrix $\boldsymbol{H}$	$\boldsymbol{H} = \boldsymbol{A} \cdot \boldsymbol{GT}$	[257800, 376+39]	[858600, 843+101]
Dimension of matrix $\boldsymbol{O}$	$\boldsymbol{O}$	[257800, 376+39+400+100] = [257800, 555]	[858600, 843+101+800+200] = [858600, 1944]
Dimension of matrix $\boldsymbol{O^T O}$	$\boldsymbol{O^T O}$	[555, 555]	[1944,1944]

The calculation of matrix $\boldsymbol{H}$ was the most computationally consuming part as large matrix multiplication of matrices $\boldsymbol{A}$, $\boldsymbol{G}$ and $\boldsymbol{T}$ was involved in its derivation. So is the calculation of matrix $\boldsymbol{O}^T\boldsymbol{O}$. Thus, we took advantage of the fast matrix multiplication ability of the graphics processing units (GPU) to accelerate these processes (Fig. S1). First, we solved the outer product of $\boldsymbol{G}$ and $\boldsymbol{T}$ by batches. We selected how many spatial bases from $\boldsymbol{G}$ to be calculated in one batch depending on the available GPU memory and the size of $\boldsymbol{G}$. Then we divided $\boldsymbol{G}$ into n groups of spatial bases as $\boldsymbol{G} = [\boldsymbol{g_1} \cdots \boldsymbol{g_n}]$ and sent batch by batch to GPU. Then we calculated in GPU the outer product of each group of spatial bases $\boldsymbol{g_i}$ and the temporal bases matrix $\boldsymbol{T}$ as $\boldsymbol{gt} = \boldsymbol{g_i} \otimes \boldsymbol{T}$. By multiplying $\boldsymbol{A}$ we derived the $\boldsymbol{h_i}$ from one batch calculation in GPU. After calculating all the batches, we derived $\boldsymbol{H} = [\boldsymbol{h_1} \cdots \boldsymbol{h_n}]$. Combining $\boldsymbol{H}$ with $\boldsymbol{\Delta OD_{ss}}$ and $\boldsymbol{OD_{drift}}$ we derived $\boldsymbol{O}$. By sending batches of $\boldsymbol{o_j}$ into GPU, we calculated matrix $\boldsymbol{o_i}^T\boldsymbol{o_j}$ and $\boldsymbol{o_i}^T\boldsymbol{y_i}$. Combining results from the batches calculation in GPU, we derived the matrix $\boldsymbol{O}^T\boldsymbol{O}$ and $\boldsymbol{O}^T\boldsymbol{Y}$.

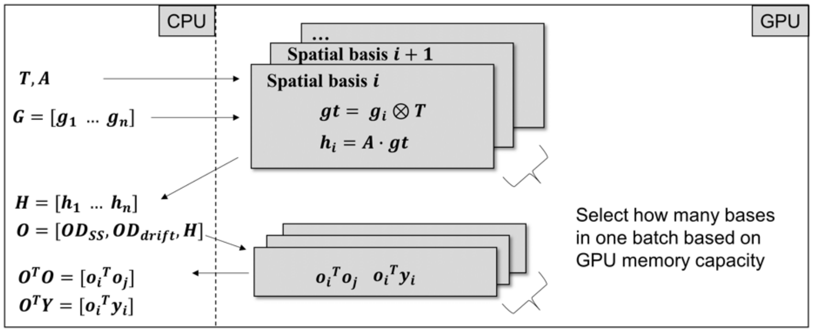

Figure S1. Diagram of data storage and calculation pipeline.

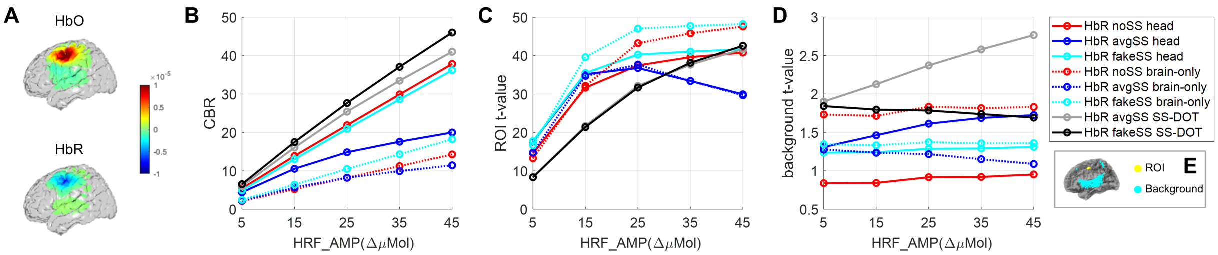

Figure S2. The results from the simulated datasets (HbR). (A) An example of the image reconstructed by fakeSS SS-DOT model (Subject #12, HRF amplitude = 25 μMol). (B) The CBR value under different HRF amplitudes for each model. (C) The average t-value in the ROI region under different HRF amplitudes for each model. (D) The average t-value in the background region under different HRF amplitudes for each model. (E) The ROI region and the background region.

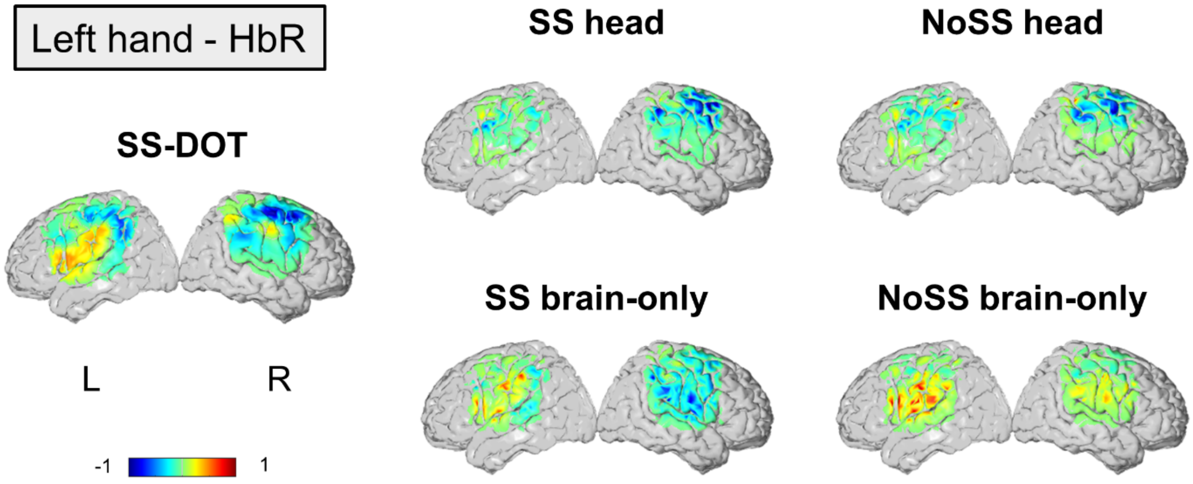

Figure S3. The group average HbR images for each model under the condition of left hand. The color bar has been normalized.

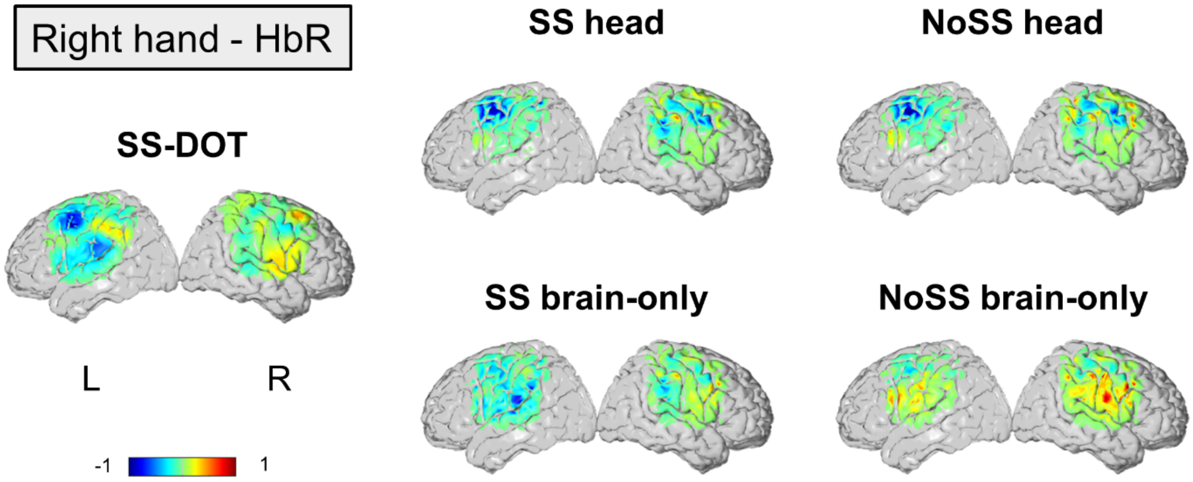

Figure S4. The group averaged HbR images for each model under the condition of right hand. The color bar has been normalized by the absolute minimum HbR value.

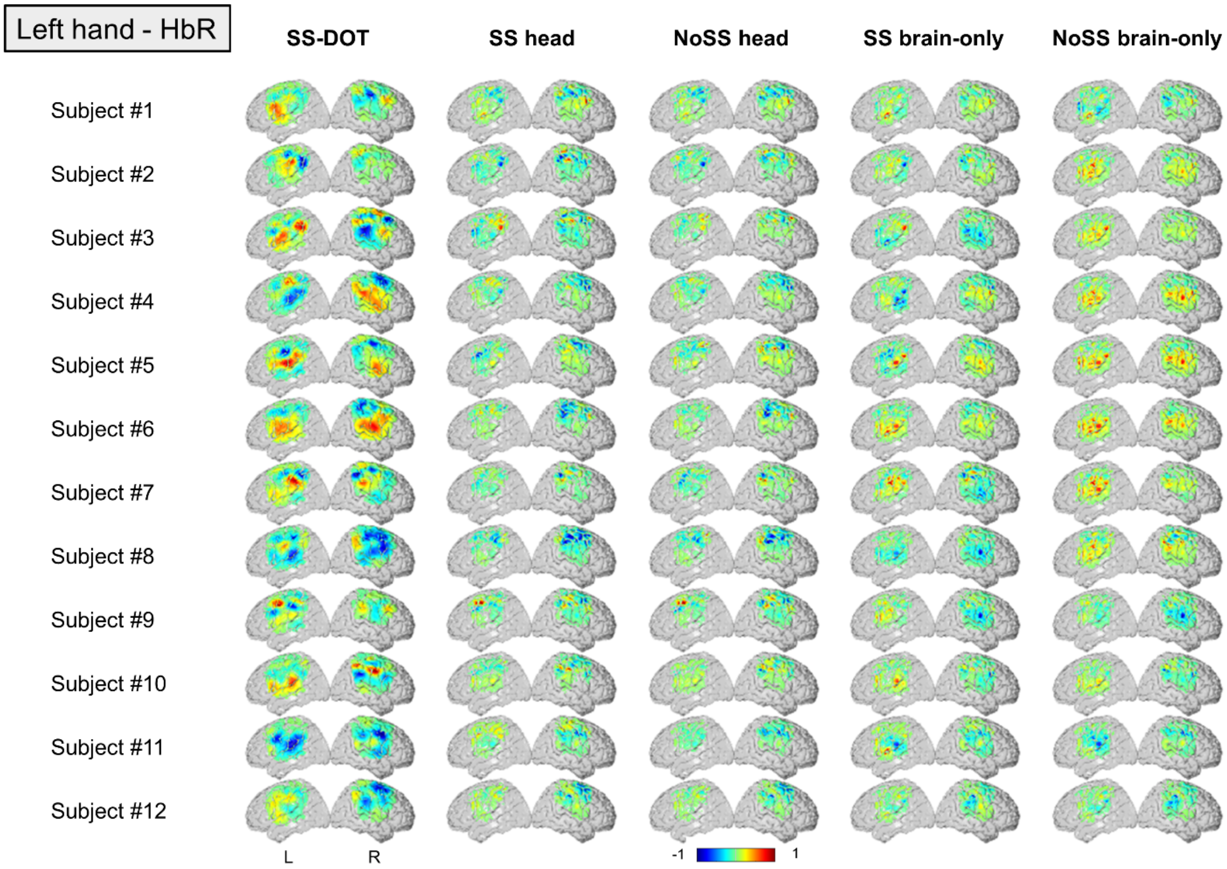

Figure S5. The subject level HbR images for each model under the condition of left hand. The color bar has been normalized.

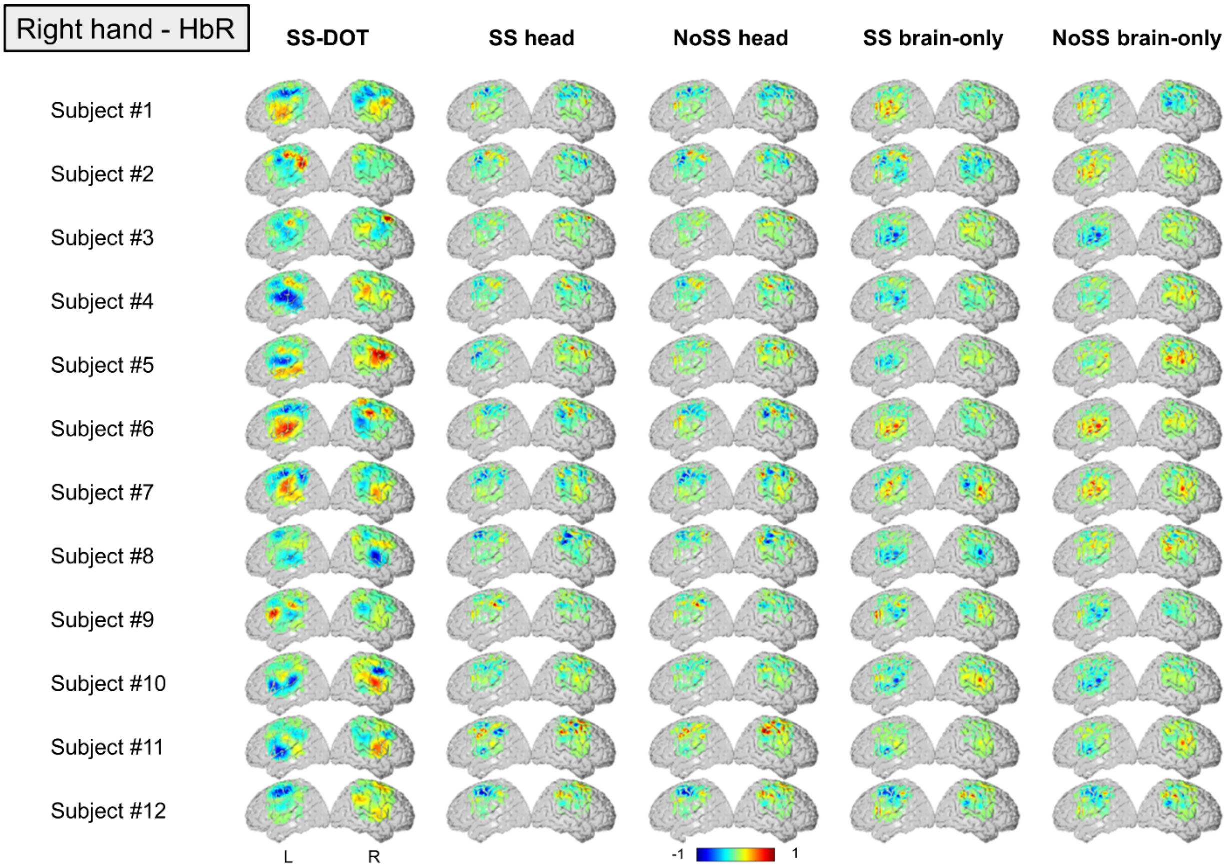

Figure S6. The subject level HbR images for each model under the condition of right hand. The color bar has been normalized by the absolute minimum HbR value.

Table S2 t-value from subject level images, CBR and LI value derived from group averaged image for each model on experimental data (HbR). The bold values are the greatest among the models.

HbR		SS-DOT	SS head	NoSS head	SS brain-only	NoSS brain-only
	t-value	2.17	2.22	2.76	2.14	**3.13**
Left hand	CBR	**1.82**	0.72	0.63	0.18	0.14
	LI	0.45	0.87	0.92	**1.00**	0.39
	t-value	1.35	1.93	2.31	2.07	**2.38**
Right hand	CBR	**2.86**	0.40	0.36	0.17	0.13
	LI	**1.00**	0.59	0.63	0.57	0.23

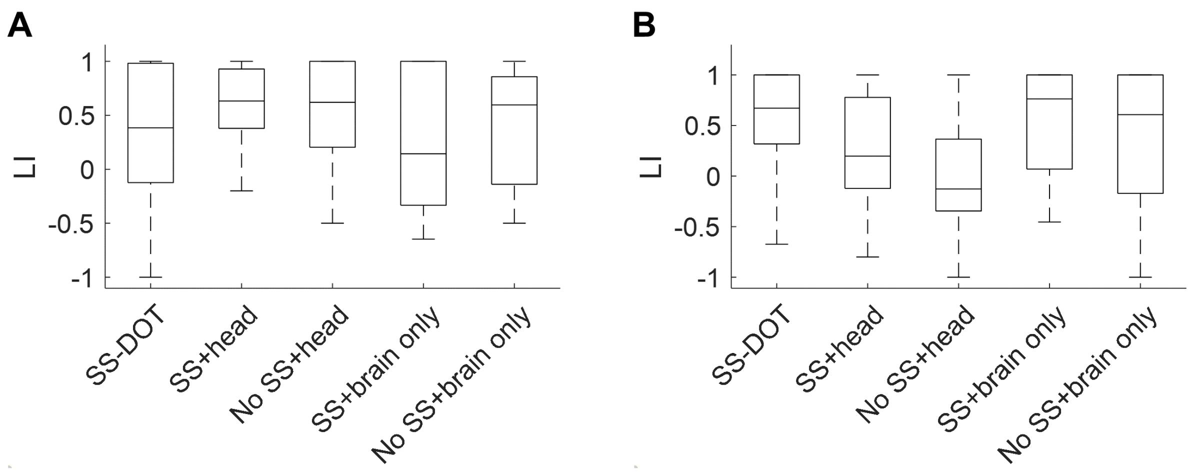

Figure S7. Laterality index (LI) of HbR for each model on the experimental dataset, for (A) left hand condition and (B) right hand condition

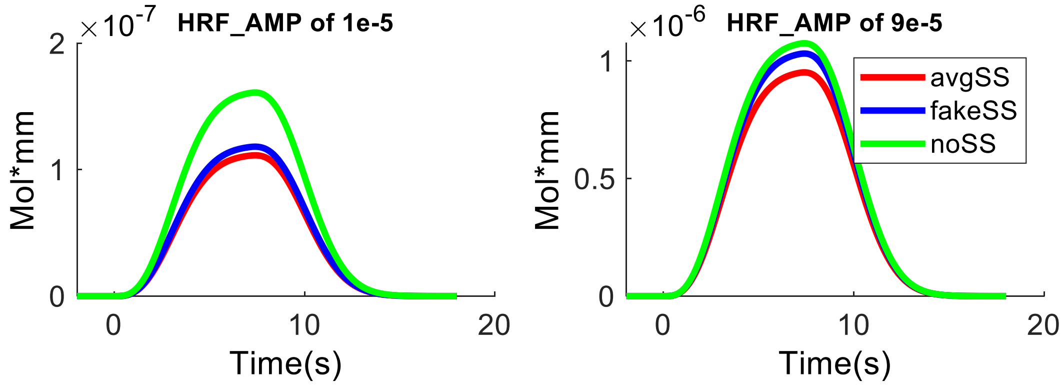

Figure S8. An example of HRFs (S4-D7) derived from 'avgSS', 'fakeSS' and 'noSS' models.

Table S3. inferential statistics of multiple comparison of LI values (left hand condition, HbO)

Group A	Group B	Lower limit	Difference	Upper limit	p-value
SS-DOT	SS+head	-32.345	-12.708	6.928	0.693
SS-DOT	no SS+head	-33.303	-13.667	5.970	0.507
SS-DOT	SS+brain only	-14.970	4.667	24.303	1.000
SS-DOT	no SS+brain only	-13.970	5.667	25.303	1.000
SS+head	no SS+head	-20.595	-0.958	18.678	1.000
SS+head	SS+brain only	-2.262	17.375	37.012	0.130
SS+head	no SS+brain only	-1.262	18.375	38.012	0.086
no SS+head	SS+brain only	-1.303	18.333	37.970	0.088
no SS+head	no SS+brain only	-0.303	19.333	38.970	0.057
SS+brain only	no SS+brain only	-18.637	1.000	20.637	1.000

Table S4. inferential statistics of multiple comparison of LI values (right hand condition, HbO)

Group A	Group B	Lower limit	Difference	Upper limit	p-value

SS-DOT	SS+head	-17.340	2.583	22.507	1.000
SS-DOT	no SS+head	-10.007	9.917	29.840	1.000
SS-DOT	SS+brain only	-12.674	7.250	27.174	1.000
SS-DOT	no SS+brain only	-9.674	10.250	30.174	1.000
SS+head	no SS+head	-12.590	7.333	27.257	1.000
SS+head	SS+brain only	-15.257	4.667	24.590	1.000
SS+head	no SS+brain only	-12.257	7.667	27.590	1.000
no SS+head	SS+brain only	-22.590	-2.667	17.257	1.000
no SS+head	no SS+brain only	-19.590	0.333	20.257	1.000
SS+brain only	no SS+brain only	-16.924	3.00	22.924	1.000

Table S5. inferential statistics of multiple comparison of LI values (left hand condition, HbR)

Group A	Group B	Lower limit	Difference	Upper limit	p-value
SS-DOT	SS+head	-25.451	-5.667	14.117	1.000
SS-DOT	no SS+head	-24.826	-5.042	14.742	1.000
SS-DOT	SS+brain only	-17.617	2.167	21.951	1.000
SS-DOT	no SS+brain only	-20.826	-1.041	18.742	1.000
SS+head	no SS+head	-19.159	0.625	20.409	1.000
SS+head	SS+brain only	-11.951	7.833	27.617	1.000
SS+head	no SS+brain only	-15.159	4.625	24.409	1.000
no SS+head	SS+brain only	-12.576	7.208	26.992	1.000
no SS+head	no SS+brain only	-15.784	4.000	23.784	1.000
SS+brain only	no SS+brain only	-22.992	-3.208	16.576	1.000

Table S6. inferential statistics of multiple comparison of LI values (right hand condition, HbR)

Group A	Group B	Lower limit	Difference	Upper limit	p-value
SS-DOT	SS+head	-9.366	10.375	30.116	1.000
SS-DOT	no SS+head	-2.658	17.083	36.824	0.151
SS-DOT	SS+brain only	-16.658	3.083	22.824	1.000
SS-DOT	no SS+brain only	-15.283	4.458	24.199	1.000
SS+head	no SS+head	-13.033	6.708	26.449	1.000
SS+head	SS+brain only	-27.033	-7.292	12.449	1.000
SS+head	no SS+brain only	-25.658	-5.917	13.824	1.000
no SS+head	SS+brain only	-33.741	-14.000	5.741	0.465
no SS+head	no SS+brain only	-32.366	-12.625	7.116	0.726
SS+brain only	no SS+brain only	-18.366	1.375	21.116	1.000

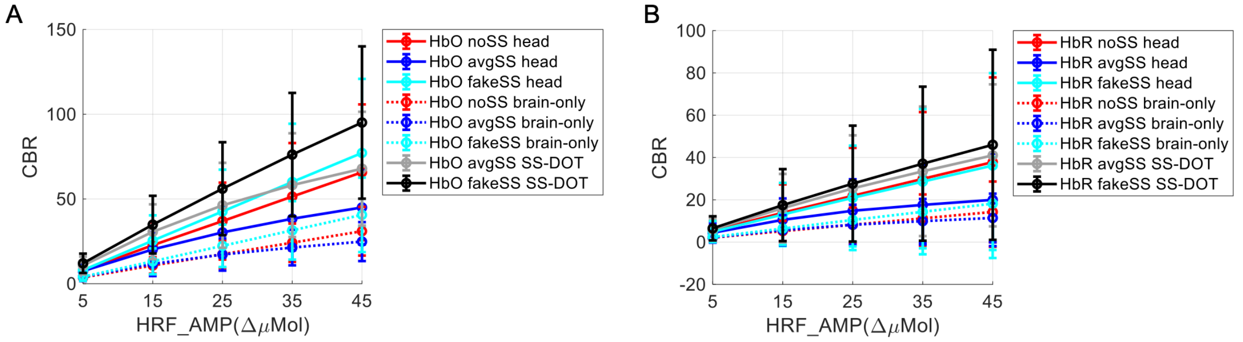

Figure S9. The mean CBR value and its standard error (the bar) under different HRF amplitudes for each model, for HbO (A) and HbR (B)

Table S7. inferential statistics of multiple comparison of CBR values (HbO), bold font indicates p value (Bonferroni corrected) greater than alpha = 0.05

**HRF_AMP ($\Delta\mu Mol$)**	**Group A**	**Group B**	**Lower limit**	**Difference**	**Upper limit**	**p-value**
5	noSS head	avgSS head	-5.575	0.193	5.962	1.000
	noSS head	fakeSS head	-6.121	-0.353	5.416	1.000
	noSS+head	no SS brain only	-1.818	3.950	9.719	0.833
	noSS+head	avg SS brain only	-2.183	3.586	9.355	1.000
	noSS+head	fake SS brain only	-2.257	3.512	9.281	1.000
	noSS+head	avgSS SS-DOT	-8.857	-3.089	2.680	1.000
	noSS+head	fakeSS SS-DOT	-10.174	-4.405	1.363	0.440
	avgSS head	fakeSS head	-6.315	-0.546	5.222	1.000
	avgSS head	no SS brain only	-2.012	3.757	9.525	1.000
	avgSS head	avg SS brain only	-2.376	3.393	9.161	1.000
	avgSS head	fake SS brain only	-2.450	3.319	9.087	1.000
	avgSS head	avgSS SS-DOT	-9.051	-3.282	2.487	1.000
	avgSS head	fakeSS SS-DOT	-10.367	-4.599	1.170	0.331
	fakeSS head	no SS brain only	-1.466	4.303	10.071	0.510
	fakeSS head	avg SS brain only	-1.830	3.939	9.707	0.845
	fakeSS head	fake SS brain only	-1.904	3.865	9.633	0.934
	fakeSS head	avgSS SS-DOT	-8.504	-2.736	3.033	1.000
	fakeSS head	fakeSS SS-DOT	-9.821	-4.053	1.716	0.724
	no SS brain only	avg SS brain only	-6.133	-0.364	5.404	1.000
	no SS brain only	fake SS brain only	-6.207	-0.438	5.330	1.000
	no SS brain only	avgSS SS-DOT	-12.807	-7.039	-1.270	**0.005**
	no SS brain only	fakeSS SS-DOT	-14.124	-8.355	-2.587	**0.000**
	avg SS brain only	fake SS brain only	-5.843	-0.074	5.695	1.000
	avg SS brain only	avgSS SS-DOT	-12.443	-6.675	-0.906	**0.010**
	avg SS brain only	fakeSS SS-DOT	-13.760	-7.991	-2.223	**0.001**
	fake SS brain only	avgSS SS-DOT	-12.369	-6.601	-0.832	**0.011**
	fake SS brain only	fakeSS SS-DOT	-13.686	-7.917	-2.149	**0.001**
	avgSS SS-DOT	fakeSS SS-DOT	-7.085	-1.317	4.452	1.000
15	noSS head	avgSS head	-14.327	2.485	19.298	1.000

	noSS head	fakeSS head	-19.502	-2.689	14.123	1.000
	noSS+head	no SS brain only	-4.837	11.976	28.788	0.670
	noSS+head	avg SS brain only	-5.740	11.073	27.885	1.000
	noSS+head	fake SS brain only	-7.297	9.516	26.328	1.000
	noSS+head	avgSS SS-DOT	-24.734	-7.922	8.891	1.000
	noSS+head	fakeSS SS-DOT	-28.931	-12.119	4.694	0.626
	avgSS head	fakeSS head	-21.987	-5.175	11.638	1.000
	avgSS head	no SS brain only	-7.322	9.491	26.303	1.000
	avgSS head	avg SS brain only	-8.225	8.588	25.400	1.000
	avgSS head	fake SS brain only	-9.782	7.031	23.843	1.000
	avgSS head	avgSS SS-DOT	-27.220	-10.407	6.405	1.000
	avgSS head	fakeSS SS-DOT	-31.417	-14.604	2.208	0.176
	fakeSS head	no SS brain only	-2.147	14.665	31.478	0.170
	fakeSS head	avg SS brain only	-3.050	13.762	30.575	0.275
	fakeSS head	fake SS brain only	-4.607	12.205	29.018	0.600
	fakeSS head	avgSS SS-DOT	-22.045	-5.233	11.580	1.000
	fakeSS head	fakeSS SS-DOT	-26.242	-9.430	7.383	1.000
	no SS brain only	avg SS brain only	-17.716	-0.903	15.910	1.000
	no SS brain only	fake SS brain only	-19.273	-2.460	14.353	1.000
	no SS brain only	avgSS SS-DOT	-36.710	-19.898	-3.085	**0.007**
	no SS brain only	fakeSS SS-DOT	-40.907	-24.095	-7.282	**<0.001**
	avg SS brain only	fake SS brain only	-18.370	-1.557	15.256	1.000
	avg SS brain only	avgSS SS-DOT	-35.807	-18.995	-2.182	**0.013**
	avg SS brain only	fakeSS SS-DOT	-40.004	-23.192	-6.379	**0.001**
	fake SS brain only	avgSS SS-DOT	-34.250	-17.438	-0.625	**0.034**
	fake SS brain only	fakeSS SS-DOT	-38.447	-21.635	-4.822	**0.002**
	avgSS SS-DOT	fakeSS SS-DOT	-21.009	-4.197	12.616	1.000
25	noSS head	avgSS head	-20.196	6.664	33.524	1.000
	noSS head	fakeSS head	-32.549	-5.689	21.171	1.000
	noSS+head	no SS brain only	-7.315	19.546	46.406	0.592
	noSS+head	avg SS brain only	-7.140	19.720	46.580	0.561
	noSS+head	fake SS brain only	-12.276	14.584	41.444	1.000
	noSS+head	avgSS SS-DOT	-36.169	-9.309	17.551	1.000
	noSS+head	fakeSS SS-DOT	-45.990	-19.130	7.730	0.670
	avgSS head	fakeSS head	-39.213	-12.353	14.507	1.000
	avgSS head	no SS brain only	-13.979	12.882	39.742	1.000
	avgSS head	avg SS brain only	-13.804	13.056	39.916	1.000
	avgSS head	fake SS brain only	-18.940	7.920	34.780	1.000
	avgSS head	avgSS SS-DOT	-42.833	-15.973	10.887	1.000
	avgSS head	fakeSS SS-DOT	-52.654	-25.794	1.066	0.074
	fakeSS head	no SS brain only	-1.626	25.235	52.095	0.090
	fakeSS head	avg SS brain only	-1.451	25.409	52.269	0.085
	fakeSS head	fake SS brain only	-6.587	20.273	47.133	0.474
	fakeSS head	avgSS SS-DOT	-30.480	-3.620	23.240	1.000

	fakeSS head	fakeSS SS-DOT	-40.301	-13.441	13.419	1.000
	no SS brain only	avg SS brain only	-26.686	0.174	27.035	1.000
	no SS brain only	fake SS brain only	-31.822	-4.962	21.899	1.000
	no SS brain only	avgSS SS-DOT	-55.715	-28.855	-1.995	**0.023**
	no SS brain only	fakeSS SS-DOT	-65.536	-38.676	-11.815	**<0.001**
	avg SS brain only	fake SS brain only	-31.996	-5.136	21.724	1.000
	avg SS brain only	avgSS SS-DOT	-55.889	-29.029	-2.169	**0.022**
	avg SS brain only	fakeSS SS-DOT	-65.710	-38.850	-11.990	**<0.001**
	fake SS brain only	avgSS SS-DOT	-50.753	-23.893	2.967	0.145
	fake SS brain only	fakeSS SS-DOT	-60.574	-33.714	-6.854	**0.003**
	avgSS SS-DOT	fakeSS SS-DOT	-36.681	-9.821	17.039	1.000
35	noSS head	avgSS head	-22.466	13.100	48.666	1.000
	noSS head	fakeSS head	-44.164	-8.598	26.968	1.000
	noSS+head	no SS brain only	-8.338	27.228	62.794	0.433
	noSS+head	avg SS brain only	-5.475	30.091	65.657	0.215
	noSS+head	fake SS brain only	-15.622	19.944	55.510	1.000
	noSS+head	avgSS SS-DOT	-42.301	-6.735	28.831	1.000
	noSS+head	fakeSS SS-DOT	-60.317	-24.751	10.815	0.763
	avgSS head	fakeSS head	-57.264	-21.698	13.868	1.000
	avgSS head	no SS brain only	-21.438	14.127	49.693	1.000
	avgSS head	avg SS brain only	-18.575	16.991	52.557	1.000
	avgSS head	fake SS brain only	-28.722	6.844	42.410	1.000
	avgSS head	avgSS SS-DOT	-55.401	-19.835	15.731	1.000
	avgSS head	fakeSS SS-DOT	-73.417	-37.851	-2.286	0.026
	fakeSS head	no SS brain only	0.259	35.825	71.391	0.046
	fakeSS head	avg SS brain only	3.123	38.689	74.255	**0.020**
	fakeSS head	fake SS brain only	-7.024	28.542	64.108	0.316
	fakeSS head	avgSS SS-DOT	-33.703	1.863	37.429	1.000
	fakeSS head	fakeSS SS-DOT	-51.720	-16.154	19.412	1.000
	no SS brain only	avg SS brain only	-32.702	2.864	38.430	1.000
	no SS brain only	fake SS brain only	-42.850	-7.284	28.282	1.000
	no SS brain only	avgSS SS-DOT	-69.528	-33.962	1.604	0.078
	no SS brain only	fakeSS SS-DOT	-87.545	-51.979	-16.413	**<0.001**
	avg SS brain only	fake SS brain only	-45.713	-10.147	25.419	1.000
	avg SS brain only	avgSS SS-DOT	-72.392	-36.826	-1.260	**0.035**
	avg SS brain only	fakeSS SS-DOT	-90.409	-54.843	-19.277	**<0.001**
	fake SS brain only	avgSS SS-DOT	-62.245	-26.679	8.887	0.492
	fake SS brain only	fakeSS SS-DOT	-80.261	-44.695	-9.129	**0.003**
	avgSS SS-DOT	fakeSS SS-DOT	-53.582	-18.017	17.549	1.000
45	noSS head	avgSS head	-22.703	20.768	64.239	1.000
	noSS head	fakeSS head	-54.875	-11.404	32.066	1.000
	noSS+head	no SS brain only	-8.684	34.787	78.258	0.322
	noSS+head	avg SS brain only	-2.566	40.905	84.376	0.089
	noSS+head	fake SS brain only	-18.338	25.132	68.603	1.000

	noSS+head	avgSS SS-DOT	-45.586	-2.115	41.356	1.000
	noSS+head	fakeSS SS-DOT	-72.817	-29.346	14.125	0.897
	avgSS head	fakeSS head	-75.643	-32.172	11.298	0.535
	avgSS head	no SS brain only	-29.452	14.019	57.490	1.000
	avgSS head	avg SS brain only	-23.334	20.137	63.608	1.000
	avgSS head	fake SS brain only	-39.107	4.364	47.835	1.000
	avgSS head	avgSS SS-DOT	-66.354	-22.883	20.588	1.000
	avgSS head	fakeSS SS-DOT	-93.585	-50.114	-6.643	**0.010**
	fakeSS head	no SS brain only	2.721	46.192	89.663	**0.026**
	fakeSS head	avg SS brain only	8.839	52.310	95.781	**0.006**
	fakeSS head	fake SS brain only	-6.934	36.537	80.008	0.226
	fakeSS head	avgSS SS-DOT	-34.182	9.289	52.760	1.000
	fakeSS head	fakeSS SS-DOT	-61.413	-17.942	25.529	1.000
	no SS brain only	avg SS brain only	-37.353	6.118	49.589	1.000
	no SS brain only	fake SS brain only	-53.126	-9.655	33.816	1.000
	no SS brain only	avgSS SS-DOT	-80.373	-36.903	6.568	0.210
	no SS brain only	fakeSS SS-DOT	-107.604	-64.134	-20.663	**<0.001**
	avg SS brain only	fake SS brain only	-59.244	-15.773	27.698	1.000
	avg SS brain only	avgSS SS-DOT	-86.491	-43.021	0.450	0.055
	avg SS brain only	fakeSS SS-DOT	-113.723	-70.252	-26.781	**<0.001**
	fake SS brain only	avgSS SS-DOT	-70.719	-27.248	16.223	1.000
	fake SS brain only	fakeSS SS-DOT	-97.950	-54.479	-11.008	**0.003**
	avgSS SS-DOT	fakeSS SS-DOT	-70.702	-27.231	16.240	1.000

Table S8. inferential statistics of multiple comparison of CBR values (HbR), bold font indicates p value (Bonferroni corrected) greater than alpha = 0.05

HRF_AMP ($\Delta\mu Mol$)	Group A	Group B	Lower limit	Difference	Upper limit	p-value
5	noSS head	avgSS head	-3.880	1.015	5.910	1.000
	noSS head	fakeSS head	-4.394	0.501	5.396	1.000
	noSS+head	no SS brain only	-1.580	3.315	8.210	0.883
	noSS+head	avg SS brain only	-1.692	3.203	8.098	1.000
	noSS+head	fake SS brain only	-1.906	2.989	7.884	1.000
	noSS+head	avgSS SS-DOT	-5.494	-0.599	4.296	1.000
	noSS+head	fakeSS SS-DOT	-5.997	-1.102	3.793	1.000
	avgSS head	fakeSS head	-5.410	-0.515	4.380	1.000
	avgSS head	no SS brain only	-2.595	2.300	7.195	1.000
	avgSS head	avg SS brain only	-2.707	2.188	7.083	1.000
	avgSS head	fake SS brain only	-2.921	1.974	6.869	1.000
	avgSS head	avgSS SS-DOT	-6.509	-1.614	3.281	1.000
	avgSS head	fakeSS SS-DOT	-7.012	-2.117	2.778	1.000
	fakeSS head	no SS brain only	-2.081	2.814	7.709	1.000
	fakeSS head	avg SS brain only	-2.193	2.702	7.597	1.000

	fakeSS head	fake SS brain only	-2.406	2.489	7.384	1.000
	fakeSS head	avgSS SS-DOT	-5.994	-1.099	3.796	1.000
	fakeSS head	fakeSS SS-DOT	-6.498	-1.603	3.292	1.000
	no SS brain only	avg SS brain only	-5.007	-0.112	4.783	1.000
	no SS brain only	fake SS brain only	-5.221	-0.326	4.569	1.000
	no SS brain only	avgSS SS-DOT	-8.809	-3.914	0.981	0.324
	no SS brain only	fakeSS SS-DOT	-9.312	-4.417	0.478	0.129
	avg SS brain only	fake SS brain only	-5.109	-0.214	4.681	1.000
	avg SS brain only	avgSS SS-DOT	-8.697	-3.802	1.093	0.394
	avg SS brain only	fakeSS SS-DOT	-9.200	-4.305	0.590	0.159
	fake SS brain only	avgSS SS-DOT	-8.483	-3.588	1.307	0.567
	fake SS brain only	fakeSS SS-DOT	-8.986	-4.091	0.804	0.236
	avgSS SS-DOT	fakeSS SS-DOT	-5.399	-0.504	4.391	1.000
15	noSS head	avgSS head	-8.067	3.342	14.751	1.000
	noSS head	fakeSS head	-10.572	0.836	12.245	1.000
	noSS+head	no SS brain only	-2.739	8.670	20.079	0.454
	noSS+head	avg SS brain only	-3.198	8.210	19.619	0.631
	noSS+head	fake SS brain only	-3.932	7.477	18.886	1.000
	noSS+head	avgSS SS-DOT	-13.574	-2.165	9.243	1.000
	noSS+head	fakeSS SS-DOT	-15.017	-3.608	7.801	1.000
	avgSS head	fakeSS head	-13.914	-2.506	8.903	1.000
	avgSS head	no SS brain only	-6.081	5.328	16.737	1.000
	avgSS head	avg SS brain only	-6.540	4.868	16.277	1.000
	avgSS head	fake SS brain only	-7.274	4.135	15.544	1.000
	avgSS head	avgSS SS-DOT	-16.916	-5.507	5.901	1.000
	avgSS head	fakeSS SS-DOT	-18.359	-6.950	4.458	1.000
	fakeSS head	no SS brain only	-3.575	7.834	19.243	0.820
	fakeSS head	avg SS brain only	-4.035	7.374	18.783	1.000
	fakeSS head	fake SS brain only	-4.768	6.640	18.049	1.000
	fakeSS head	avgSS SS-DOT	-14.411	-3.002	8.407	1.000
	fakeSS head	fakeSS SS-DOT	-15.854	-4.445	6.964	1.000
	no SS brain only	avg SS brain only	-11.868	-0.460	10.949	1.000
	no SS brain only	fake SS brain only	-12.602	-1.193	10.216	1.000
	no SS brain only	avgSS SS-DOT	-22.244	-10.835	0.573	0.082
	no SS brain only	fakeSS SS-DOT	-23.687	-12.278	-0.870	**0.023**
	avg SS brain only	fake SS brain only	-12.143	-0.734	10.675	1.000
	avg SS brain only	avgSS SS-DOT	-21.785	-10.376	1.033	0.120
	avg SS brain only	fakeSS SS-DOT	-23.228	-11.819	-0.410	**0.035**
	fake SS brain only	avgSS SS-DOT	-21.051	-9.642	1.767	0.217
	fake SS brain only	fakeSS SS-DOT	-22.494	-11.085	0.324	0.066
	avgSS SS-DOT	fakeSS SS-DOT	-12.852	-1.443	9.966	1.000
25	noSS head	avgSS head	-9.545	7.053	23.651	1.000
	noSS head	fakeSS head	-15.650	0.947	17.545	1.000
	noSS+head	no SS brain only	-2.910	13.688	30.285	0.260

	noSS+head	avg SS brain only	-2.923	13.675	30.273	0.262
	noSS+head	fake SS brain only	-5.190	11.408	28.006	0.816
	noSS+head	avgSS SS-DOT	-20.124	-3.526	13.072	1.000
	noSS+head	fakeSS SS-DOT	-22.370	-5.772	10.826	1.000
	avgSS head	fakeSS head	-22.704	-6.106	10.492	1.000
	avgSS head	no SS brain only	-9.963	6.634	23.232	1.000
	avgSS head	avg SS brain only	-9.976	6.622	23.220	1.000
	avgSS head	fake SS brain only	-12.243	4.355	20.953	1.000
	avgSS head	avgSS SS-DOT	-27.177	-10.580	6.018	1.000
	avgSS head	fakeSS SS-DOT	-29.423	-12.825	3.773	0.408
	fakeSS head	no SS brain only	-3.858	12.740	29.338	0.426
	fakeSS head	avg SS brain only	-3.870	12.728	29.326	0.428
	fakeSS head	fake SS brain only	-6.137	10.461	27.058	1.000
	fakeSS head	avgSS SS-DOT	-21.072	-4.474	12.124	1.000
	fakeSS head	fakeSS SS-DOT	-23.317	-6.719	9.879	1.000
	no SS brain only	avg SS brain only	-16.610	-0.012	16.586	1.000
	no SS brain only	fake SS brain only	-18.877	-2.280	14.318	1.000
	no SS brain only	avgSS SS-DOT	-33.812	-17.214	-0.616	**0.034**
	no SS brain only	fakeSS SS-DOT	-36.057	-19.459	-2.861	**0.008**
	avg SS brain only	fake SS brain only	-18.865	-2.267	14.331	1.000
	avg SS brain only	avgSS SS-DOT	-33.800	-17.202	-0.604	**0.035**
	avg SS brain only	fakeSS SS-DOT	-36.045	-19.447	-2.849	**0.008**
	fake SS brain only	avgSS SS-DOT	-31.532	-14.934	1.663	0.132
	fake SS brain only	fakeSS SS-DOT	-33.778	-17.180	-0.582	**0.035**
	avgSS SS-DOT	fakeSS SS-DOT	-18.843	-2.245	14.353	1.000
35	noSS head	avgSS head	-8.651	12.314	33.279	1.000
	noSS head	fakeSS head	-19.669	1.296	22.261	1.000
	noSS+head	no SS brain only	-2.290	18.675	39.640	0.143
	noSS+head	avg SS brain only	-0.970	19.995	40.960	0.079
	noSS+head	fake SS brain only	-5.382	15.583	36.549	0.521
	noSS+head	avgSS SS-DOT	-24.538	-3.572	17.393	1.000
	noSS+head	fakeSS SS-DOT	-28.143	-7.178	13.787	1.000
	avgSS head	fakeSS head	-31.984	-11.019	9.947	1.000
	avgSS head	no SS brain only	-14.604	6.361	27.326	1.000
	avgSS head	avg SS brain only	-13.284	7.681	28.646	1.000
	avgSS head	fake SS brain only	-17.696	3.269	24.234	1.000
	avgSS head	avgSS SS-DOT	-36.852	-15.887	5.079	0.462
	avgSS head	fakeSS SS-DOT	-40.457	-19.492	1.473	0.099
	fakeSS head	no SS brain only	-3.586	17.379	38.344	0.251
	fakeSS head	avg SS brain only	-2.265	18.700	39.665	0.142
	fakeSS head	fake SS brain only	-6.677	14.288	35.253	0.854
	fakeSS head	avgSS SS-DOT	-25.833	-4.868	16.097	1.000
	fakeSS head	fakeSS SS-DOT	-29.438	-8.473	12.492	1.000
	no SS brain only	avg SS brain only	-19.645	1.320	22.286	1.000

	no SS brain only	fake SS brain only	-24.057	-3.092	17.874	1.000
	no SS brain only	avgSS SS-DOT	-43.212	-22.247	-1.282	**0.027**
	no SS brain only	fakeSS SS-DOT	-46.818	-25.853	-4.888	**0.004**
	avg SS brain only	fake SS brain only	-25.377	-4.412	16.553	1.000
	avg SS brain only	avgSS SS-DOT	-44.533	-23.568	-2.603	**0.014**
	avg SS brain only	fakeSS SS-DOT	-48.138	-27.173	-6.208	**0.002**
	fake SS brain only	avgSS SS-DOT	-40.121	-19.156	1.809	0.116
	fake SS brain only	fakeSS SS-DOT	-43.726	-22.761	-1.796	**0.021**
	avgSS SS-DOT	fakeSS SS-DOT	-24.570	-3.605	17.360	1.000
45	noSS head	avgSS head	-7.259	17.850	42.959	0.677
	noSS head	fakeSS head	-23.472	1.637	26.745	1.000
	noSS+head	no SS brain only	-1.545	23.564	48.673	0.091
	noSS+head	avg SS brain only	1.294	26.403	51.512	**0.030**
	noSS+head	fake SS brain only	-5.477	19.632	44.741	0.377
	noSS+head	avgSS SS-DOT	-28.297	-3.189	21.920	1.000
	noSS+head	fakeSS SS-DOT	-33.299	-8.191	16.918	1.000
	avgSS head	fakeSS head	-41.322	-16.213	8.895	1.000
	avgSS head	no SS brain only	-19.395	5.714	30.823	1.000
	avgSS head	avg SS brain only	-16.555	8.553	33.662	1.000
	avgSS head	fake SS brain only	-23.327	1.782	26.891	1.000
	avgSS head	avgSS SS-DOT	-46.147	-21.038	4.070	0.232
	avgSS head	fakeSS SS-DOT	-51.149	-26.041	-0.932	**0.034**
	fakeSS head	no SS brain only	-3.181	21.927	47.036	0.168
	fakeSS head	avg SS brain only	-0.342	24.766	49.875	0.057
	fakeSS head	fake SS brain only	-7.113	17.995	43.104	0.647
	fakeSS head	avgSS SS-DOT	-29.934	-4.825	20.284	1.000
	fakeSS head	fakeSS SS-DOT	-34.936	-9.827	15.281	1.000
	no SS brain only	avg SS brain only	-22.269	2.839	27.948	1.000
	no SS brain only	fake SS brain only	-29.041	-3.932	21.177	1.000
	no SS brain only	avgSS SS-DOT	-51.861	-26.752	-1.644	**0.026**
	no SS brain only	fakeSS SS-DOT	-56.863	-31.755	-6.646	**0.003**
	avg SS brain only	fake SS brain only	-31.880	-6.771	18.337	1.000
	avg SS brain only	avgSS SS-DOT	-54.700	-29.592	-4.483	**0.008**
	avg SS brain only	fakeSS SS-DOT	-59.702	-34.594	-9.485	**0.001**
	fake SS brain only	avgSS SS-DOT	-47.929	-22.820	2.288	0.121
	fake SS brain only	fakeSS SS-DOT	-52.931	-27.823	-2.714	**0.016**
	avgSS SS-DOT	fakeSS SS-DOT	-30.111	-5.002	20.107	1.000
